# Subjective regulation success mediates the influence of neurofeedback performance on mood in the absence of training effects

**DOI:** 10.3389/fpsyg.2026.1743425

**Published:** 2026-07-02

**Authors:** M. Klöbl, S. D. Robinson, L. R. Silberbauer, M. B. Reed, A. Sahl, D. Gomola, A. Hahn, R. Lanzenberger

**Affiliations:** 1 Department of Psychiatry and Psychotherapy, Medical University of Vienna, Vienna, Austria; 2 Comprehensive Center for Clinical Neurosciences and Mental Health, Medical University of Vienna, Vienna, Austria; 3 Department of Biomedical Imaging and Image-Guided Therapy, Medical University of Vienna, Vienna, Austria; 4 Centre for Advanced Imaging, University of Queensland, St Lucia, QLD, Australia

**Keywords:** fMRI, mood, neurofeedback, placebo, specificity

## Abstract

**Background:**

Functional magnetic resonance imaging neurofeedback (fMRI NF) is gaining popularity as an experimental treatment for psychiatric and neurologic disorders. However, concerns that fMRI NF acts as a placebo rather than through operant conditioning principles, as known for electroencephalography NF, have largely been disregarded.

**Methods:**

To examine these concerns, 25 healthy individuals underwent training to downregulate activity in the amygdala and subgenual anterior cingulate cortex, and to upregulate both regions concurrently as a bidirectional control condition, across six sessions in a randomized, single-blind, within-subject design.

**Results:**

While we found no consistent changes in brain activation or training effects across participants, subjective ratings of regulation success mediated the effect of brain activation and objectively achieved regulation on mood changes for both brain regions and regulation directions. Quantified emotional valence and arousal of the regulation strategies had no detectable influence at any stage.

**Conclusion:**

Our findings suggest predominantly non-specific effects of fMRI NF on mood changes in healthy individuals, which are mediated by the impression of subjective regulation success—at least in the absence of training effects. While the results partially support concerns about the specificity of fMRI NF, further research is required to determine how these findings generalize across different training protocols and patient populations.

## Introduction

Neurofeedback (NF) is a largely experimental treatment that uses operant conditioning and reinforcement learning to achieve neural self-regulation. While NF has risen in popularity (Wider et al., 2024), based on the application of electroencephalography (EEG) NF in the treatment of attention-deficit/hyperactivity disorder (ADHD), claims of lacking specificity and placebo effects have also increased (Kober et al., 2023). Well-controlled functional magnetic resonance imaging (fMRI) NF studies or studies targeting disorders other than ADHD increasingly report no superiority over sham conditions (Holtmann et al., 2011; Lam et al., 2022; Schabus et al., 2017; Thibault et al., 2018b; Neurofeedback Collaborative Group, 2021; Neurofeedback Collaborative Group, 2023). It is therefore crucial to investigate whether EEG NF’s alleged lack of specificity translates to fMRI NF.

In fMRI NF, the treatment regimen often involves up- or downregulating activity in a specific brain region linked to the targeted disorder over multiple sessions (Linhartová et al., 2019). However, regimens vary widely, even within the same condition (Thibault et al., 2016), including the brain regions (Compère et al., 2023b; Hamilton et al., 2016; Zhao et al., 2023; Zweerings et al., 2018) and their intended regulation direction (Zhao et al., 2023; Zotev et al., 2018). This calls into question the specific roles of the target brain regions and their neural activity. Furthermore, side effects such as decreased positive affect resulting from exhausting training can further obscure the specificity of NF (Johnston et al., 2010; Klöbl et al., 2020; Zahn et al., 2019). Despite the potentially strenuous training, it has been shown that just a few fMRI NF sessions can be sufficient, such that, e.g., only three sessions yielded clinically and statistically significant symptom improvements in patients with post-traumatic stress disorder (Zotev et al., 2018; Zhao et al., 2023).

Some evidence also suggests that the individual regulation strategies may be important for successful intervention (Hasslinger et al., 2020) or may contribute to symptom improvement beyond the regulation of the target brain regions (Klöbl et al., 2023). However, there is contrary evidence that up- and downregulation of slow cortical potentials can be achieved without changing strategy (Siniatchkin et al., 2000), and fMRI NF may even be feasible without the use of strategies at all (Ramot et al., 2016). Moreover, while it seems clinically reasonable to provide patients with initial strategies that can be further developed, detailed instructions might actually have detrimental effects (Zotev et al., 2018; Sepulveda et al., 2016). Consequently, it remains unclear whether the specific regulatory strategies used influence the success of NF.

Despite growing criticism, the origins of the purported placebo effect in NF remain insufficiently investigated (Aggensteiner, 2021). To address this gap, we examined whether neural, behavioral, or subjective factors primarily drive NF-related mood changes. Direct influence of NF-related changes in brain activation suggests a neural origin. If mood changes primarily depend on the feedback received, then reinforcement is more important. Lastly, if the subjective impression of regulation success predominantly influences mood changes, this points toward an non-specific or placebo effect (Trimmer et al., 2013; Mehler et al., 2018).

With the growing use of NF as an experimental treatment for depression (Khaleghi et al., 2025; Linhartová et al., 2019; Xia et al., 2024; Trambaiolli et al., 2021), it is important to assess the suitability of potential target regions. The amygdala is one of the most frequently targeted brain regions in fMRI NF (Linhartová et al., 2019), while the subgenual anterior cingulate cortex (sgACC) is mostly targeted in deep brain stimulation (Drobisz and Damborská, 2019) and plays important roles in the effects of ketamine (Alexander et al., 2021) and targeted transcranial magnetic stimulation (Alexander et al., 2021) in antidepressant treatment. Since hyperactivity in both regions, as well as increased functional connectivity between them, has been linked to depression (Hamani et al., 2011; Grogans et al., 2022; Connolly et al., 2013), we trained participants to downregulate their respective activity using fMRI NF. This hyperactivation also suggests that simultaneous upregulation of both regions might be possible using negative affective strategies. Among previous studies, comparisons to active rather than passive control conditions were associated with smaller effect sizes (Trambaiolli et al., 2021). While this indicates the influence of non-specific treatment factors, it also underscores the importance of active control conditions. Therefore, we used concurrent upregulation of the amygdala and sgACC as a bidirectional, active control condition. As further hypothesized factors influencing NF performance, we assessed the content of the regulation strategies and initial mood (Nadler et al., 2010).

## Methods and materials

The investigations presented here were part of the optimization phase of a larger project assessing the suitability of fMRI NF as a therapeutic option for treatment-resistant depression. Therefore, the sample size was estimated based on clinical depression ratings. Previous study samples yielded a mean score of 30.48 ± 5.74 (mean ± standard deviation) on the Hamilton Depression Rating Scale. Assuming a parallel-group design with a 50% reduction in symptom severity in the active group, given the generally large effect sizes reported for NF (Khaleghi et al., 2025), and a 10% reduction in the sham group, Cohen’s d was estimated at 0.60, corresponding to 19 participants at an alpha level of 0.05 and 80% power. This estimate was also used in the optimization phase.

### Study procedure and subjects

**Figure 1:**
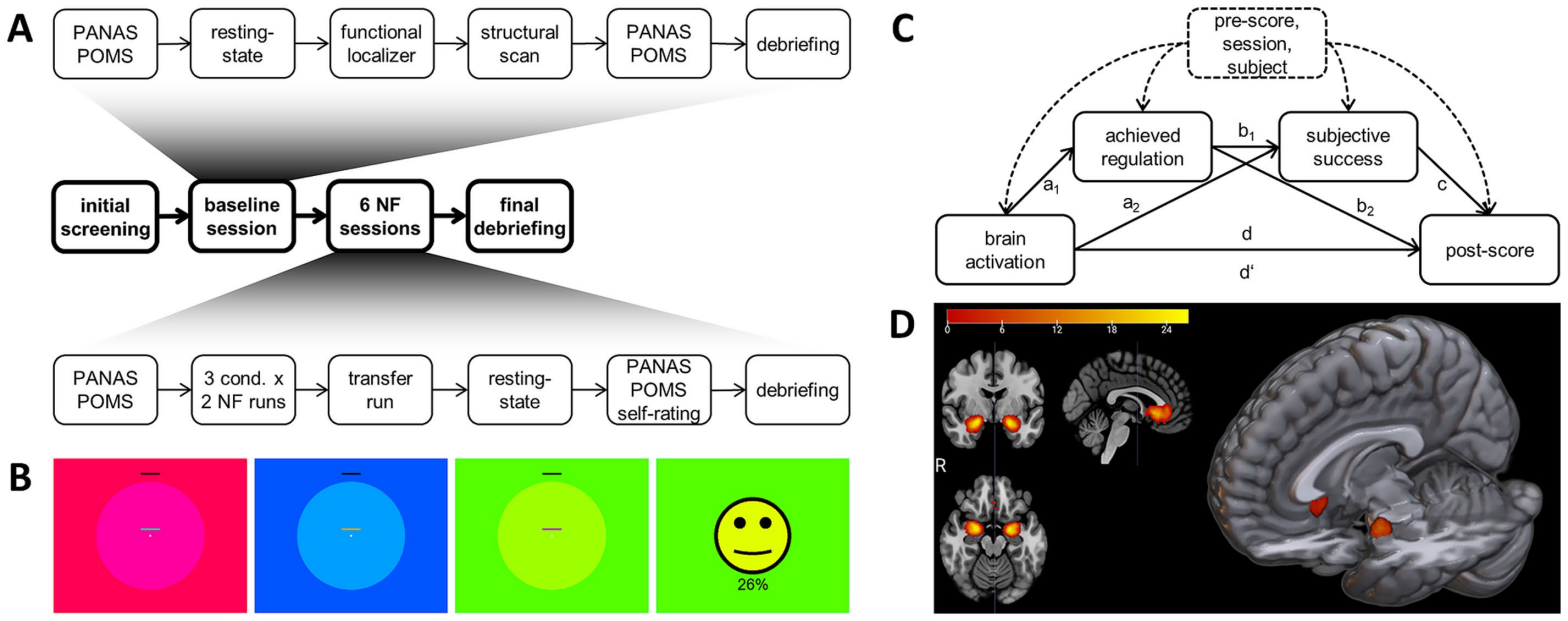
Overview of the study, functional localization, feedback, and mediation modeling. **(A)** Sequence of sessions (middle, bold) with details on the baseline (top) and neurofeedback (NF) sessions (bottom). **(B)** Example feedback screens for the color-coded conditions. The colored bar moved and indicated the currently achieved regulation. The black bar at the top indicated 100% regulation. An additional reward in the form of a smiley face with the achieved regulation was given if subjects reached at least 10%. Each color represented one of the three *regulatory* conditions (downregulation of the amygdala or the subgenual anterior cingulate cortex, or upregulation of both regions together). The assignment was random but stayed the same within a subject. **(C)** Serial mediation model. Arrows indicate effect directions; dashed elements indicate covariates/random effects. The model tested whether activation of the target regions influenced the achieved regulation, that is, the feedback the subjects received, which, in turn, affected the perceived subjective success quantified via self-ratings. The effects influence each other and the post-session mood scores. All effects were adjusted for individual subject, session, and pre-session mood scores. **(D)** Heat map of the parts of the subgenual anterior cingulate cortex and bilateral amygdalae as selected via the functional localizer. For visual purposes, the 3D rendering only shows the parts selected for at least 15 subjects. PANAS: Positive and Negative Affect Schedule, POMS: Profile of Mood States.

The study followed a single-blind, randomized within-subject design. Healthy subjects had to be 18–35-year olds and received an initial screening, including a detailed explanation of the study procedures. Inclusion required the absence of current psychiatric disorders, as assessed using the Structured Clinical Interviews for Axis I and II Disorders (SCID I/II) according to the *Diagnostic and Statistical Manual of Mental Disorders, 4th Edition, Text Revision (DSM-IV-TR)*. A score below 7 on the 17-item Hamilton Depression rating scale quantitatively ruled out relevant depressive symptoms. After inclusion, subjects underwent one baseline MRI session (Supplementary material: Baseline session) followed by six fMRI NF sessions (median ± interquartile range [IQR] = 3 ± 3 days between NF sessions), which is well within the range of clinical fMRI NF trials. While many proof-of-concept studies use only one or two sessions (Khaleghi et al., 2025), a recent review reported the benefits of additional training sessions on depressive symptoms and neuropsychological functioning (Xia et al., 2024). Participation ended with a final debriefing (Figure 1A). The study was conducted in accordance with the Declaration of Helsinki, was approved by the institutional review board of the Medical University of Vienna (number 1937/2016), and all subjects gave written informed consent.

### Instructions

Participants were informed that the goal of the study was to gain volitional control over three brain regions that are involved in emotion processing by developing mental strategies that reliably produce the desired feedback. No example strategies were provided to avoid an additional bias (Sepulveda et al., 2016). To determine whether a strategy was effective, subjects were asked to apply it for at least one full regulation block (see “Neurofeedback run” section). They were informed about the randomized color-coding of the regions (red, green, and blue; Figure 1B) and that the colors had no further meaning.

### Regulation conditions

Unbeknownst to the subjects, the three brain regions to control implied downregulation of the sgACC, downregulation of the bilateral amygdalae, and simultaneous upregulation of both regions as a bidirectional control condition (Sorger et al., 2019). Bidirectional training is considered one of the strictest control conditions in fMRI NF. It allows for assessing the specificity of the regulatory direction: If the NF-induced changes in neural activation are necessary for the observed psychometric or behavioral changes, there should be a positive relationship with the active but not with the control (i.e., inverse) direction. As previous NF studies have shown, amygdala and sgACC activity can be volitionally increased or decreased without external stimuli, providing the ideal foundation for a bidirectional control condition (Hamilton et al., 2007; Hamilton et al., 2011; Watve et al., 2024).

### Neurofeedback sessions

The NF sessions began with participants completing the Positive and Negative Affect Schedule (PANAS) and the Profile of Mood States (POMS) before entering the scanner. Subjects underwent two consecutive NF runs of each of the three conditions. The order of conditions was randomized between subjects and sessions. The association between conditions and colors was also randomized within subjects, but remained the same once assigned. The last NF run was followed by a transfer run containing all three conditions in random order. MRI acquisition ended with a resting-state scan (not presented here). After scanning, subjects provided visual analog scale (VAS) ratings of their subjective performance for the three colors (i.e., conditions) and again answered the PANAS and POMS. In a short debriefing, subjects reported on their strategies and subjective experiences (Figure 1A).

#### Neurofeedback run

Each NF run consisted of twelve 20-s regulation blocks with continuous feedback separated by baseline blocks of equal duration. OpenNFT handled all calculations, and the feedback display (Koush et al., 2017) with adaptations as described in the Supplementary material under “Online processing and OpenNFT extensions.” Feedback was displayed as a bar in the center of the screen that participants were instructed to move up toward a target bar. If the currently applied strategy caused activity to change in the wrong direction, the bar would drop below the center of the screen. In addition, the screen color brightness corresponded to the NF signal, with increased brightness indicating more positive feedback. This additional feedback mechanism was implemented because some participants in the pilot study found it easier to apply their NF strategies with their eyes closed or without looking at the feedback (Klöbl et al., 2020). This was, however, not explicitly mentioned in the task explanation, and participants in this study were not encouraged to close their eyes. The mechanism was implemented so that they would at least receive a vague response to their current strategy, even with their eyes closed. During the baseline, only a white fixation dot was shown at the center of the screen with no changes in screen color or brightness. After blocks with sufficiently strong regulation, a smiley face with the degree of smiling corresponding to the amount of regulation and the achieved percentage of the maximum achievable regulation, as estimated by OpenNFT, was shown for 2 s. This value was calculated as the median from 6 s after the beginning of the regulation block to its end. A 10% change in the intended direction relative to the median of the preceding baseline block with the same delay was considered sufficient. Continuous and intermittent feedback were provided due to their complementary advantages (Klöbl et al., 2020). We employed an adapted version of OpenNFT [Supplementary material: Online processing and OpenNFT extensions (Koush et al., 2017)] for online processing and feedback presentation.

#### Transfer run

No feedback was provided during the transfer run, which comprised six consecutive blocks of each condition, without bars or brightness changes. As in NF training, the regulation blocks were separated by baseline periods, and the conditions were identified by their assigned colors. The order of conditions was randomized, and the timing was the same as for the NF runs. Subjects were instructed to apply the strategy or strategies that yielded the most consistent positive feedback for the respective color during each of the six regulation blocks.

### Analysis of strategies

The strategies reported by the subjects as “successful” after each NF session were rated independently for valence (i.e., quality of the emotional affect) and arousal on a seven-point Likert scale by the first author (MK), a psychologist (DG), and a psychology student (AS). For further analysis, the mean of the three independent ratings was used. Further details on the employed strategies are provided in the “Strategies” section in the Supplementary material.

### Statistical modeling

For fMRI acquisition, preprocessing, modeling, and extraction of the target brain activation, please refer to the “Statistical modeling” section in the Supplementary material.

#### Models

We tested the influence of the pre-session emotional state, as well as the valence and arousal of the developed strategies, using model Equation 1. Session and run effects provided insight into whether the subjects gained control over their brain activation. The effects of brain activation, achieved regulation, and the subjective impression of regulation success, as well as the strategies’ valence and arousal, on mood changes were tested using model Equation 2. To quantify the overall influence of the received feedback, subjective regulation, valence, and arousal, we averaged these variables across conditions per session in model Equation 2. However, we used the original activation values because their influence may depend on the brain region. To cover potential time-varying influences of feedback and strategies on mood changes, linear and square models with and without interaction were compared, and the one with the lowest Watanabe-Akaike Information Criterion (WAIC) was selected (Konicar et al., 2021). Model selection was performed to avoid overly complex models. The models were first run without valence and arousal ratings since these were missing when subjects did not report successful strategies. They were subsequently rerun with valence and arousal ratings added. For the rationale of the model structure and further explanations on the variables, see “Model structure” in the Supplementary material.

Variables and notation: act: brain activation extracted from the offline processed fMRI data, reg: achieved regulation (the shown NF signal), suc: subjective rating of regulation success (reported via visual analog scales), ses: session, run: first or second of the two NF runs performed per session and condition, poms: POMS score (either depression [dep], anger [ang], fatigue [fat], or vigor [vig]), val: valence, arous: arousal, sub: subject, col.: color of condition, colAsgmt: assignment of all colors, ord: position of condition, ordAsgmt: order of conditions, pre: pre-session scores, post: post-session scores, acc: subgenual anterior cingulate cortex, amy: amygdalae, ctrl: control condition, avg.: average over conditions, /: both variables or operations were modeled separately, []: models with and without the term in the brackets were tested.


$$\begin{array}{c} \mathrm{act}/\mathrm{reg}\sim {\mathrm{ses}}^{\left[2\right]}+\mathrm{run}+{\mathrm{dep}}_{\mathrm{pre}}+{\mathrm{ang}}_{\mathrm{pre}}+{\mathrm{fat}}_{\mathrm{pre}}+{\mathrm{vig}}_{\mathrm{pre}}\\ +[\mathrm{val}+\text{arous}+](1+{\mathrm{ses}}^{\left[2\right]}+\mathrm{run}|\mathrm{sub})\\ +(1|\mathrm{col})+(1|\mathrm{ord}) \end{array}$$ (1)

$$\begin{array}{c} {\text{poms}}_{\text{post}}\sim {\mathrm{ses}}^{\left[2\right]}+/*(\begin{array}{l} {\text{poms}}_{\mathrm{pre}}+{\mathrm{act}}_{\mathrm{acc}}+{\mathrm{act}}_{\mathrm{amy}}+{\mathrm{act}}_{\text{ctrl}}\\ +{\mathrm{reg}}_{\mathrm{avg}}+{\mathrm{suc}}_{\mathrm{avg}}[+{\mathrm{val}}_{\mathrm{avg}}+{\text{arous}}_{\mathrm{avg}}] \end{array})\\ +(1+{\mathrm{ses}}^{\left[2\right]}|\mathrm{sub})+(1|\text{colAsgmt})+(1|\text{ordAsgmt}) \end{array}$$ (2)

The achieved regulation (represented as the NF signal) was mathematically derived from brain activation, with variations arising only from differences between fast online and more extensive offline processing. Thus, as an intermediate step before mediation analysis, only the dependence of subjective ratings of regulation success on the NF signal and pre-session mood required to be investigated using model Equation 3.


$$\begin{array}{c} \mathrm{suc}\sim {\mathrm{ses}}^{\left[2\right]}+/*({\mathrm{dep}}_{\mathrm{pre}}+{\mathrm{ang}}_{\mathrm{pre}}+{\mathrm{fat}}_{\mathrm{pre}}+{\mathrm{vig}}_{\mathrm{pre}}+\mathrm{reg})\\ +(1+{\mathrm{ses}}^{\left[2\right]}|\mathrm{sub})+(1|\mathrm{col})+(1|\mathrm{ord}) \end{array}$$ (3)

Potential effect paths from brain activation through achieved regulation and subjective regulation success, driving mood changes, were modeled as fixed-effects serial mediation according to Equation 4 (Figure 1C). Covariates comprised a subject factor, linear and squared session number, and pre-session mood scores. *Post hoc* tests of simple causal mixed mediation effects, also taking repeated measures into account, were carried out according to model Equation 5 using the “mediation” package. The models were again adjusted for pre-session scores and the linear and squared session number as covariates. For exploratory analyses of the potential moderated mediation of subjective success by achieved regulation, refer to the supplementary “Moderated mediation analyses” methods and results sections and the supplementary discussion section.


$${\text{poms}}_{\text{post}}\sim \mathrm{act}+\mathrm{reg}+\mathrm{suc}+{\mathrm{ses}}^{2}+{\text{poms}}_{\mathrm{pre}}+\mathrm{sub}$$ (4)

$${\text{poms}}_{\text{post}}\sim \mathrm{reg}+\mathrm{suc}+{\mathrm{ses}}^{2}+{\text{poms}}_{\mathrm{pre}}+(1+{\mathrm{ses}}^{2}|\mathrm{sub})$$ (5)

#### Software and model implementation

Statistical modeling and testing of relationships between variables without explicit mediation was conducted via Bayesian inference using Integrated Nested Laplace Approximations in R (INLA; Rue et al., 2009; r-inla.org). Since accounting for the randomization process, that is, random assignment of colors to the conditions, and runs within sessions resulted in rather complex models, Bayesian inference provided the benefit of increased robustness to overfitting compared to frequentist approaches (Flores et al., 2022). Bounded dependent variables (achieved regulation, success self-ratings, POMS/PANAS scores) were modeled using beta distributions with logit link functions in generalized linear mixed models. Otherwise, the identity link function, being equivalent to linear mixed models, was used. Bounded independent variables were logit-transformed.

Serial mediation analyses were run using the “process” macro (Hayes, 2022) (version 4.3) and *post hoc* analyses in the “mediation” package (Imai et al., 2010) (version 4.5.0) in R. Due to limitations of “process,” serial mediation was tested on the fixed effects model named “Model 6” in the macro. Furthermore, the crossed random effects for condition color and order could also not be modeled in the simple mixed mediation analyses due to limitations of the “mediation” package. For all mediation analyses, bounded dependent and independent variables were logit-transformed.

We used the POMS as the primary tool for quantifying mood due to its more fine-grained differentiation comprising scales for “depression,” “anger,” “fatigue,” and “vigor.” All analyses were primarily performed with the data collected during the NF training runs. Separate analyses of the transfer run data were performed using the same models.

### Testing

The 95% credible interval (CI) reported by INLA not covering 0 can be interpreted as roughly equivalent to a two-sided *p*-value smaller than 0.05.

For the serial mediation analysis, we ran “process” with 5,000 bootstrap samples, standardized, bootstrapped, and type 3 heteroscedasticity-corrected confidence intervals (CIs). Sine similarity provided dependency-adjusted multiplicity correction (Klöbl et al., 2023).

#### Results

##### Demographics

Of the 27 enrolled subjects, 20 completed the study, and 2 dropped out before the first NF session. Partially available data of the remaining five subjects, who underwent NF sessions, were included in the analyses whenever possible. Subjects were 23.62 ± 3.96 years old (median ± interquartile range [IQR]), and 15 were identified as female.

##### Influence of pre-training mood and strategies on achieved regulation

**Figure 2:**
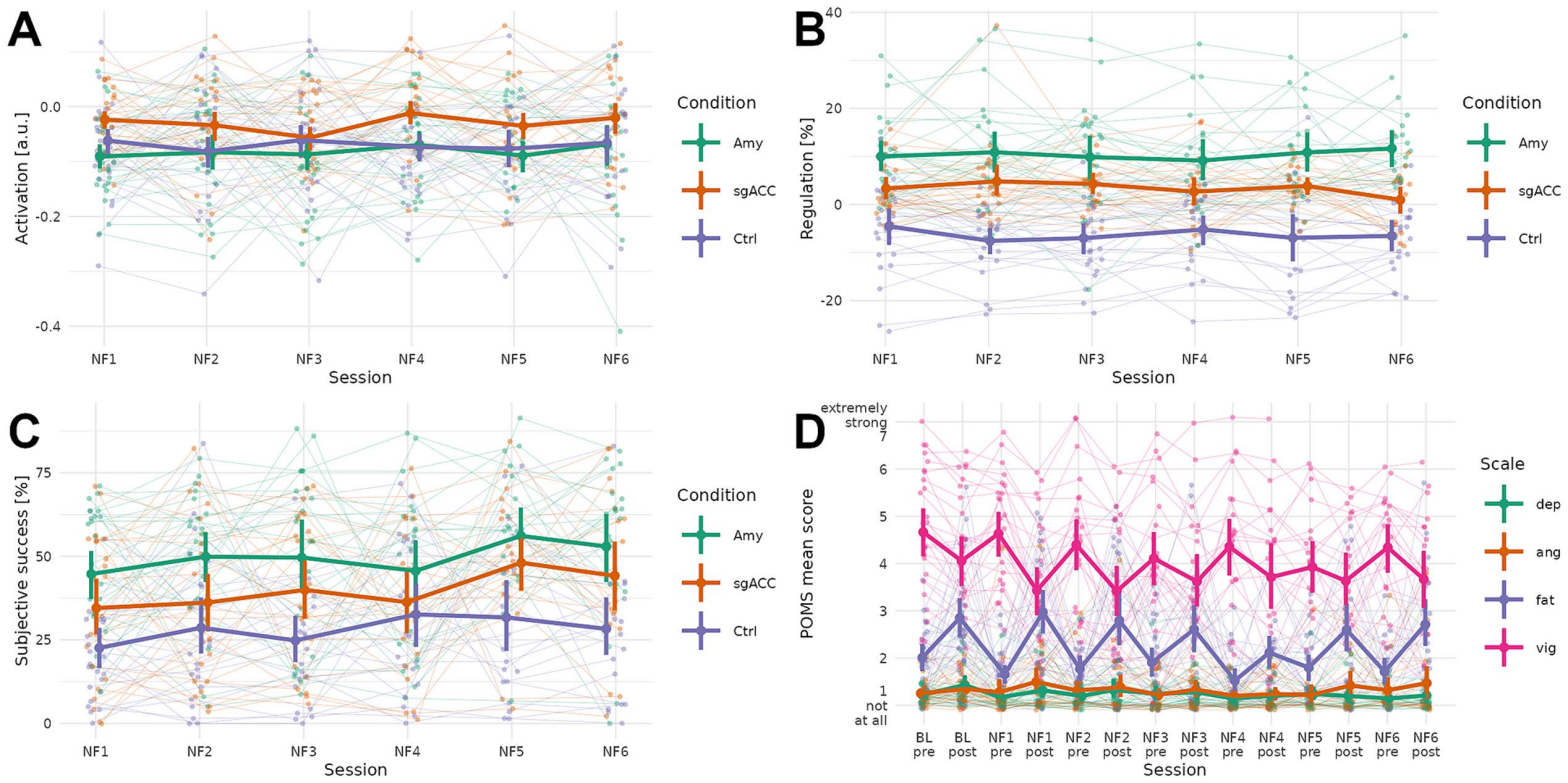
Individual and mean (with 95% confidence intervals) variable time courses. **(A)** Neurofeedback brain activation (beta estimates) after offline processing. **(B)** Achieved regulation as shown to the participants (the neurofeedback signal from online processing). **(C)** Subjective success as rated via visual analog scales. **(D)** Scales of the Profile of Mood States (POMS) questionnaire. BL: baseline session, NF: neurofeedback session, Amy: amygdala, sgACC: subgenual anterior cingulate cortex, Ctrl: control condition, dep: depression, ang: anger, fat: fatigue, vig: vigilance.

A relevant regulation effect was observed only for the achieved amygdala downregulation, that is, the CI did not cover zero ( β=0.183 , CI=[0.051,0.315] ). However, this was not the case for brain activation or the other conditions. CIs for session number also covered zero, indicating no robust change over time. Model comparison yielded lower WAIC measures for quadratic influences of session number on both amygdala activation and regulation. There was a negative influence of pre-training fatigue on amygdala activation ( β=−0.017 , CI=[−0.032,−0.003] ) and pre-training vigor on the brain activation ( β=−0.020 , CI=[−0.033,−0.007] ) and the achieved regulation ( β=−0.036 , CI=[−0.069,−0.003] ) during the control condition. The CIs for valence and arousal covered zero, indicating no relevant influence on brain activation or achieved regulation. See Figure 2 and Supplementary Figure S1 for the variable time courses. Repeating the analyses with the transfer run brain activation data did not replicate the above findings, but showed pre-session anger positively influencing brain activation during the control condition ( β= 0.017, CI=[0.005,0.028] ). See Supplementary Table S2 for details.

##### Subjective ratings of achieved regulation

All models for the subjective ratings of the achieved regulation favored a linear influence of session number without interactions over a quadratic one but only showed reliable influences of the actually achieved regulation (amygdala: β=2.375 , CI=[1.573,3.166] , sgACC: β=3.137 , CI=[1.875,4.384] , control: β=2.455 , CI=[1.203,3.715] ). See Supplementary Table S3 for details.

##### Concurrent influence of brain activation and regulation on mood changes

Model comparison favored linear models over quadratic ones with additional interaction terms for depression and fatigue. For all scales, the pre-session scores positively influenced the post-session ones (depression: β=0.493 , CI=[0.305,0.672] , anger: β=0.425 , CI=[0.303,0.544] , fatigue: β=0.303 , CI=[0.074,0.522] , vigor: β=0.445 , CI=[0.242,0.636] ). The pre-session depression score ( β=−0.086 , CI=[−0.153,−0.017] ) as well as the achieved regulation ( β=−1.414 , CI=[−2.699,−0.130] ) increasingly reduced the post-session depression score over time. Post-session anger decreased with subjective regulation success ( β=−0.334 , CI=[−0.495,−0.169] ). Post-session fatigue increased with the achieved regulation ( β=5.000 , CI=[0.798,9.175] ). However, the negative interaction between achieved regulation and session number shows that the influence of the achieved regulation was reduced over sessions and eventually became negative ( β=−2.146 , CI=[−3.738,−0.551] ). There was also a negative influence of the subjective regulation success ( β=−0.381 , CI=[−0.676,−0.092] ). Post-session vigor decreased with amygdala activation ( β=−2.762 , CI=[−5.022,−0.506] ) but increased with activation during the control condition ( β=3.200 , CI=[0.613,5.777] ) and average subjective regulation success ( β=0.284 , CI=[0.133,0.434] ). There were no reliable influences of valence and arousal of the regulation strategies. An overview is presented in Table 1.

**Table 1:** Overview of the influences on the post-session subscales of the profile of mood states.

Subscale	Variable	Estimate	95% CI
Depression	Pre-session depression	0.493	0.305, 0.672
	Pre-session depression * Session number	−0.086	−0.153, −0.017
	Average achieved regulation * Session number	−1.414	−2.699, −0.130
Anger	Pre-session anger	0.425	0.303, 0.544
	Average subjective regulation success	−0.334	−0.495, −0.169
Fatigue	Pre-session fatigue	0.303	0.074, 0.522
	Average achieved regulation	5.000	0.798, 9.175
	Average achieved regulation * Session number	−2.146	−3.738, −0.551
	Average subjective regulation success	−0.381	−0.676, −0.092
Vigor	Pre-session vigor	0.445	0.242, 0.636
	Amygdala activation	−2.762	−5.022, −0.506
	Control activation (amygdala + sgACC)	3.200	0.613, 5.777
	Average subjective regulation success	0.284	0.133, 0.434

CI: credible interval, sgACC: subgenual anterior cingulate cortex.

Overall, higher achieved regulation and perceived regulation success led to better mood scores after training. Changes of brain activation in the intended direction in particular increased post-training vigor.

##### Mediation analysis

Results for at least uncorrected significant mediation paths are provided in Table 2. For the three regulation conditions and four scales, sine similarity estimated an adjusted significance threshold of *p* < 0.0048, corresponding to 99.52% confidence intervals (CI_95_: 95%-CI, CI_99_: 99.52%-CI). Brain activation during the control condition showed a significant positive total effect on vigor ( d=5.093 , ${\mathrm{CI}}_{95}=[0.886,9.300]$ , ${\mathrm{CI}}_{99}=[-1.026,11.212]$ ) and negative total effect on fatigue ( d=−6.714 , ${\mathrm{CI}}_{95}=[-12.754,-0.674]$ , ${\mathrm{CI}}_{99}=[-15.497,2.070]$ ). These results confirm the positive influence of brain activation during the control condition on vigor in the previous section. Significant indirect effects for the full mediation path from brain activation, through achieved regulation and subjective regulation success, to the post-session scores were found from amygdala downregulation to post-session depression ( $a_{1}*b_{1}*c=0.051$ , ${\mathrm{CI}}_{95}=[0.008,0.143]$ , ${\mathrm{CI}}_{99}=[-0.010,0.197]$ ) and fatigue( $a_{1}*b_{1}*c=0.043$ , ${\mathrm{CI}}_{95}=[0.001,0.120]$ , ${\mathrm{CI}}_{99}=[-0.016,0.163]$ ; stronger deactivation implying lower scores), sgACC activation to vigor ( $a_{1}*b_{1}*c=-0.035$ , ${\mathrm{CI}}_{95}=[-0.082,-0.012],$ ${\mathrm{CI}}_{99}=[-0.117,-0.002]$ ; stronger deactivation implying higher scores), and both regions during the control condition to fatigue ( $a_{1}*b_{1}*c=-0.037$ , ${\mathrm{CI}}_{95}=[-0.105,-0.008]$ , ${\mathrm{CI}}_{99}=[-0.134,0.005]$ ; stronger activation implying lower scores) and vigor ( $a_{1}*b_{1}*c=0.029$ , ${\mathrm{CI}}_{95}=[0.005,0.084]$ , $CI_{99}=[-0.005,0.114]$ ; stronger activation implying higher scores). Finally, there was also a significant direct effect of brain activation during the control condition on vigor ( $d^{\prime }=4.925$ , ${\mathrm{CI}}_{95}=[0.269,9.58]$ , ${\mathrm{CI}}_{99}=[-1.847,11.696]$ ; stronger activation implying higher scores). See Table 2 for details. Only the negative indirect effect of sgACC downregulation on vigor was significant for the 99.52% confidence intervals.

**Table 2:** Significant results of serial mediation fixed effects analyses.

Condition	Subscale	Effect	Estimate	95% CI	99.52% CI	*p*-value	Activation direction	Regulation/success direction
Amygdala downregulation	Depression	Indirect	0.051	0.008, 0.143	−0.010, 0.197		↑	↓
	Fatigue	Indirect	0.043	0.001, 0.120	−0.016, 0.163		↑	↓
sgACC downregulation	Vigor	Indirect	−0.035	−0.082, −0.012	−0.117, −0.002		↓	↑
Control (amygdala + sgACC upregulation)	Fatigue	Total	−6.714 (−0.323)	−12.754, −0.674	−15.497, 2.070	0.0297	↓	↓
		Indirect	−0.037	−0.105, −0.008	−0.134, 0.005		↓	↓
	Vigor	Total	5.093 (0.251)	0.886, 9.300	−1.026, 11.212	0.0181	↑	↑
		Direct	4.925 (0.243)	0.269, 9.580	−1.847, 11.696	0.0384	↑	↑
		Indirect	0.029	0.005, 0.084	−0.005, 0.114		↑	↑

No *p*-values are provided for indirect effects due to potentially inappropriate *t*-tests, and CIs are provided instead. CIs that do not cover 0 can be interpreted as significant. Indirect effects are provided as completely standardized. Completely standardized effect estimates for total and direct effects are given in parentheses. CI, Confidence interval; sgACC, Subgenual anterior cingulate cortex. The indirect effects describe the full mediation path from brain activation, through achieved regulation and subjective regulation success, to the post-session scores (*a*_1_ * *b*_1_ * *c* in Figure 1C). The total effect summarizes all paths from brain activation to pot-session mood (d in Figure 1C). The direct effect describes only the immediate influence of brain activation on post-session mood (d’ in Figure 1C).

Simple mixed mediation models confirmed the path from achieved regulation to subjective regulation success for the five significant serial indirect fixed mediation effects (amygdala/depression: $b_{1}*c=-0.98$ , p<2e−16 ; amygdala/fatigue: $b_{1}*c=-1.06$ , p=0.0016 ; sgACC/vigor: $b_{1}*c=0.83$ , p=0.0004 ; control/fatigue: $b_{1}*c=-0.96$ , p<2e−16 ; control/vigor: $b_{1}*c=0.57$ , p=0.0052 ). Except for sgACC downregulation and anger, subjective regulation success mediated the influence of the achieved regulation on post-session mood for all conditions and scales. At an adjusted significance threshold for these seven models of *p* < 0.0081, the effects of sgACC downregulation on depression ( $b_{1}*c=-0.83$ , p=0.0016 ) and fatigue ( $b_{1}*c=-0.79$ , p=0.0080 ), as well as the control condition on depression ( $b_{1}*c=-0.72$ , p=0.0012 ) remained significant. The mediation by subjective regulation success did not survive multiplicity correction for the influence of amygdala downregulation ( $b_{1}*c=-0.74$ , p=0.0300 ) and the control condition ( $b_{1}*c=-0.66$ , p=0.0160 ) on anger, as well as for the amygdala downregulation on vigor ( $b_{1}*c=0.58$ , p=0.0300 ). Details are provided in Table 3.

**Table 3:** Significant simple mixed mediation effects.

Condition	Subscale	Estimate	95% CI	*p*-value
Amygdala downregulation	Depression*	−0.98	−1.60, −0.45	< 2e-16
	Anger	−0.74	−1.49, −0.07	0.0300
	Fatigue*	−1.06	−1.84, −0.38	0.0016
	Vigor	0.58	0.06, 1.15	0.0300
sgACC downregulation	Depression	−0.83	−1.50, −0.28	0.0016
	Fatigue	−0.79	−1.61, −0.17	0.0080
	Vigor*	0.83	0.33, 1.46	0.0004
Control (amygdala + sgACC upregulation)	Depression	−0.72	−1.35, −0.24	0.0012
	Anger	−0.66	−1.38, −0.10	0.0160
	Fatigue*	−0.96	−1.75, −0.34	< 2e-16
	Vigor*	0.57	0.14, 1.16	0.0052

* Confirmatory tests of serial mediation results. sgACC: Subgenual anterior cingulate cortex. The estimates correspond to the path from achieved regulation over subjective regulation success to post-session mood scores (b_1_*c in Figure 1C). The effect estimates are for the CI, which indicates the confidence interval here.

The POMS total mood disturbance also displayed significant serial mediation for the amygdala ( $a_{1}*b_{1}*c=0.048$ , ${\mathrm{CI}}_{95}=[0.009,0.141]$ ) and sgACC ( $a_{1}*b_{1}*c=0.033$ , ${\mathrm{CI}}_{95}=[0.007,0.087]$ ) downregulation and simple mediation for all three conditions (amygdala: $b_{1}*c=-0.60$ , p<2e−16 ; sgACC: $b_{1}*c=-0.57$ , p<2e−16 ; control: $b_{1}*c=-0.46$ , p=0.0008 ; Supplementary results).

In summary, regardless of brain region or direction of regulation, the achieved regulation was associated with improved mood after training. The self-rated subjective regulation success widely mediated this effect. Furthermore, upregulation in the amygdala and activation of the sgACC were directly linked to increased vigor.

## Discussion

In this study, we demonstrated that mood changes following fMRI NF training were largely mediated by the subjective impression of regulation success, that is, the self-rating of the subjects’ performance. We found this relationship between mood changes and subjective regulation success for the sgACC and the bilateral amygdala, for both upregulation and downregulation. Although the full serial mediation from brain activation to feedback and self-rating of regulatory success was only significant for the effects of sgACC downregulation on anger, self-ratings significantly mediated feedback across the majority of the conditions and subscales, and also after multiplicity adjustment. The absence of neural changes and training effects limits our findings to the relationship between within- and between-subjects fluctuations in NF performance. However, similar effects for both upregulation and downregulation of the same brain regions and the mediation by subjective self-ratings of success support the claim that non-specific effects of fMRI NF play a role in modulating mood.

### The origin of neurofeedback’s mood-enhancing effects

Several EEG NF studies that reported no superiority over sham treatment have attributed symptom improvements to psychotherapeutic, placebo, or non-specific effects (Neurofeedback Collaborative Group, 2021; Neurofeedback Collaborative Group, 2023; Lansbergen et al., 2011; Perreau-Linck et al., 2010; Schabus et al., 2017). A critical review identified the suggestive power of neuroscience and highly technologized therapy as a potential root of NF-related treatment effects in ADHD (Thibault et al., 2018b). While this notion is reasonable, it neither explains why treatment effects may still lack in such a suggestive environment nor why they might correlate with likely NF-induced neural changes (Canterberry et al., 2013; Li et al., 2013; Liu et al., 2022; Tinaz et al., 2022). Our finding that the subjective impression of success mediates the effect on mood could fill these gaps.

An earlier study already suggested that the rewarding experience of successful regulation holds therapeutic potential, as it found strong and comparable antidepressant effects when upregulating emotion-related regions, as well as visual regions in the control condition (Mehler et al., 2018). Similar to our amygdala results, patients in that study achieved successful upregulation without significant changes over five sessions. Our mediation results, which were present irrespective of whether successful regulation was achieved (amygdala) or not (sgACC, control condition), could indicate that believing in successful regulation is sufficient for positive affective effects and that true regulatory success is unnecessary. This notion is supported by a previous study using sham NF of varying magnitude, displaying the alleged neural effects of an intravenous placebo (Peciña et al., 2018; Peciña et al., 2023). In that study, mood changes of the participants with depression were assessed after each infusion and sham NF display. Higher sham NF magnitude and higher expectation rating resulted in higher mood ratings. These findings on treatment expectations align with the positive effects of believing one will receive a real rather than a sham neurostimulation treatment (Fassi et al., 2024). In conclusion, the present study extends previous findings of strong affective effects of active control and sham NF by highlighting that objective feedback and, to some extent, the belief in successful self-regulation mediates neural effects.

Only in the control condition did brain activation directly influence post-session vigor and, to a lesser extent, fatigue (total effect only); however, both effects did not survive multiplicity correction. Since the control condition implied upregulating two brain regions simultaneously, this finding might be related to the link between the global fMRI signal and vigilance (Wong et al., 2013). A higher global signal amplitude at baseline would indicate lower vigilance and impede further volitional upregulation. Lower vigor and higher fatigue post-session could reflect vigilance (Johnson and Gurubhagavatula, 2023).

In summary, improved mood through the impression of success and control might complement the previously proposed suggestive nature of NF as a scientifically based high-tech therapy. However, brain activity itself might influence vigilance, which can be seen as an aspect of affect (Johnston et al., 2010; Klöbl et al., 2020; Zahn et al., 2019).

### Influences of strategies and mood on neurofeedback

Developing regulation strategies is essential for personalized NF and presumably influences training outcomes (Sepulveda et al., 2016; Hasslinger et al., 2020). In a previous study, we found significant correlations between the improvement in autism-related symptoms and fMRI brain activation during the application of NF regulation strategies, but not between symptom improvements and changes in slow cortical potentials, which constituted the NF target (Klöbl et al., 2023). Based on this observation, we had hypothesized that the content of the regulation strategies might contribute to the therapeutic success.

In the current study, we used valence and arousal to quantify the strategies reported as effective by participants. However, we found no reliable influence of valence or arousal on changes in brain activation or changes in brain activation or on achieved regulation. This approach is limited by the task to trying different strategies during the NF training. Still, we did not find an influence on transfer runs, where subjects were instructed to apply only their most successful strategies.

Although a link between pre-training mood and NF performance seems reasonable (Nadler et al., 2010), our results, particularly regarding brain activation, were partly contradictory. On the one hand, fatigue reduced amygdala activation. Conversely, vigor reduced brain activation during the control condition. As fatigue and vigor are negatively correlated (see Supplementary Table S1), and the control condition included amygdala activation, these results are inconclusive. Further targeted investigations are necessary to determine whether these are false-positive findings or whether vigor mainly influenced sgACC activation during the control condition.

### Methodological challenges and the future of (fMRI) neurofeedback

It is essential for the therapeutic application of NF that the discussion and investigation of its specificity and scientific validity are conducted according to scientific standards. In addition to liberal control conditions, the incorrect application of operant conditioning principles, which form the theoretical basis of NF, was also identified as a potential source of overestimated effectiveness (Pigott et al., 2021). A major concern is the use of adaptive thresholds that reward a constant number of trials, ultimately penalizing participants who perform above these thresholds (Pigott et al., 2017). This could explain the lack of effects in studies using such adaptive thresholds. If subjects are rewarded in a fixed number of trials regardless of their performance, they experience an equal amount of success. This supports our interpretation that NF influences mood through the perception of control and success, also in the absence of true neural changes (Arnold et al., 2013; Lansbergen et al., 2011, Perreau-Linck et al., 2010; Schönenberg et al., 2017). However, we cannot yet rule out neural or further behavioral influences. Since NF is based on the close relationship between neural changes and feedback/reward according to the principles of operant conditioning, future studies could probe these potential influences by modulating this relationship.

Currently, EEG NF is most widely used for the treatment of ADHD and post-traumatic stress disorder (Thibault et al., 2018a; Thibault and Raz, 2017; Ding et al., 2026). While EEG NF showed improvements in domains beyond primary PTSD symptoms, such as depression and anxiety, meta-analyses indicate that the pattern of reduced or even vanishing effect sizes in actively controlled and fMRI NF studies compared to passively controlled ones repeats here (Berman et al., 2025; Hong and Park, 2022; Askovic et al., 2023; Voigt et al., 2024). fMRI NF studies often already have stricter control conditions than EEG studies, resulting in more specific outcomes (Sorger et al., 2019; Thibault et al., 2018a). In its application as antidepressant therapy, for instance, fMRI NF has demonstrated significant relationships between amygdala activation and symptom improvements (Khaleghi et al., 2025; Young et al., 2017; Compère et al., 2023a). While previous literature has shown that apparently NF-related improvements in depressive symptoms (Mehler et al., 2018) or mood (Peciña et al., 2023; Peciña et al., 2018) are not necessarily specific to the target brain region or veridical NF, these relationships deserve further attention. Thus, further exploration of (fMRI) NF’s mechanisms of action (fMRI) could benefit future therapeutic developments. In particular, suggested communalities between biofeedback and psychotherapy (Frank et al., 2010; Kleinbub et al., 2020) and the patient-therapist relationship (Neurofeedback Collaborative Group, 2023; Norcross and Wampold, 2011) might constitute promising avenues to pursue.

### Limitations

This study was originally planned to include parallel groups with a yoked sham condition, but this had to be changed due to resource limitations. These limitations, along with our findings, led to the termination of the overarching project assessing the suitability of fMRI NF as a therapeutic option for treatment-resistant depression. As an optimization step in a larger project, this study was not pre-registered. For future investigations, pre-registering subprojects may facilitate the publication process and reduce publication bias. Although the sample size was within the range of comparable fMRI NF studies, it was estimated for the final phase of the project rather than the optimization phase. Furthermore, conceptualizing this study as an optimization step makes it inherently exploratory. These factors increase the risk of low power and false positive findings.

We did not observe any robust training effects, i.e., improvements over time or changes in brain activation, across the entire group of participants. The individual trajectories may have varied too much to produce reliable group results. The amygdala may have been successfully downregulated in the initial sessions, with no further improvement, or this effect may have been general rather than NF-specific. Furthermore, the within-subjects design with three conditions may not have enabled participants to focus sufficiently on one condition, as would have been the case in the original parallel-groups design. The lack of a training effect means that the observed mediation effects are limited to fluctuations within and between subjects, and systematic neural and time effects are excluded.

This study was single-blinded, as it used a purely within-subjects design, and the existence of a control condition was not disclosed to participants. Double-blinding would prevent experimenters from noticing whether subjects performed consistently differently in a particular condition. However, since participants found the control condition most challenging (Figure 2C), we cannot entirely rule out a residual bias, although it is likely negligible given our results. Our serial mediation model implicitly assumes that the more comprehensive offline processing of the fMRI data provides a closer approximation of the true brain activity than online processing. However, the ground truth is unknown. Furthermore, since the “process” macro (Hayes, 2022) is not intended for serial longitudinal mediation, we utilized fixed-effects models adjusted for repeated measures and conducted *post hoc* simple mixed-effects longitudinal mediation modeling.

Lastly, our study is limited to brain regions involved in emotion regulation and to healthy individuals. The mood scores could therefore be affected by floor and ceiling effects. Performance in patients might also differ due to motivational reasons or increased ranges for regulating dysfunctional brain activation patterns (Haugg et al., 2021).

## Conclusion

Our findings suggest that, at least in the absence of systematic neural changes and training effects, the positive effects of fMRI NF on the mood of healthy individuals are mediated by the subjective impression of successful self-regulation, independent of the brain region and the direction of regulation. Therefore, we infer that in this scenario, a sense of success, rather than a neural origin, governs the emotional effects of fMRI NF. While our study supports the claim that non-specific effects play an important role in the affective properties of fMRI NF, further investigations are necessary to assess to what extent the observed mediation by subjective success generalizes to trials with robust neural changes and training effects.

## Data Availability

The data supporting the conclusions of this article will be made available upon reasonable request to the corresponding author.

## References

[ref1] Aggensteiner P. M. (2021). Editorial: the complexity of neurofeedback and control of placebo effects. J. Am. Acad. Child Adolesc. Psychiatry 60, 811–812. doi: 10.1016/j.jaac.2021.05.008 34048885

[ref2] Alexander L. Jelen L. A. Mehta M. A. Young A. H. (2021). The anterior cingulate cortex as a key locus of ketamine's antidepressant action. Neurosci. Biobehav. Rev. 127, 531–554. doi: 10.1016/j.neubiorev.2021.05.003, 33984391

[ref3] Arnold L. E. Lofthouse N. Hersch S. Pan X. Hurt E. Bates B. . (2013). EEG neurofeedback for ADHD: double-blind sham-controlled randomized pilot feasibility trial. J. Atten. Disord. 17, 410–419. doi: 10.1177/1087054712446173, 22617866 PMC3939717

[ref4] Askovic M. Soh N. Elhindi J. Harris A. W. F. (2023). Neurofeedback for post-traumatic stress disorder: systematic review and meta-analysis of clinical and neurophysiological outcomes. Eur. J. Psychotraumatol. 14:2257435. doi: 10.1080/20008066.2023.2257435, 37732560 PMC10515677

[ref5] Berman D. E. Cowansage K. P. Bellanti D. M. Nair R. Boyd C. C. Beech E. H. . (2025). Systematic review and meta-analysis of neurofeedback training efficacy and neural mechanisms in the treatment of posttraumatic stress disorder. Front. Neurosci. 19:1658652. doi: 10.3389/fnins.2025.1658652, 41415692 PMC12708586

[ref6] Canterberry M. Hanlon C. A. Hartwell K. J. Li X. Owens M. Lematty T. . (2013). Sustained reduction of nicotine craving with real-time neurofeedback: exploring the role of severity of dependence. Nicotine Tob. Res. 15, 2120–2124. doi: 10.1093/ntr/ntt122, 23935182 PMC3819983

[ref7] Compère L. Siegle G. J. Lazzaro S. Strege M. Canovali G. Barb S. . (2023a). Real-time functional magnetic resonance imaging neurofeedback training of amygdala upregulation increases affective flexibility in depression. J. Psychiatry Neurosci. 48, E232–e239. doi: 10.1503/jpn.220208, 37339817 PMC10281719

[ref8] Compère L. Siegle G. J. Riley E. Lazzaro S. Strege M. Pacoe E. . (2023b). Enhanced efficacy of Cbt following augmentation with amygdala rtfmri neurofeedback in depression. J. Affect. Disord. 339, 495–501. doi: 10.1016/j.jad.2023.07.063, 37459978 PMC10530481

[ref9] Connolly C. G. Wu J. Ho T. C. Hoeft F. Wolkowitz O. Eisendrath S. . (2013). Resting-state functional connectivity of subgenual anterior cingulate cortex in depressed adolescents. Biol. Psychiatry 74, 898–907. doi: 10.1016/j.biopsych.2013.05.036, 23910949 PMC4103629

[ref10] Ding P. Tan L. Pan H. Gong A. Nan W. Fu Y. (2026). The lack of neurofeedback training regulation guidance and process evaluation may be a source of controversy in post-traumatic stress disorder-neurofeedback research: a systematic review and statistical analysis. Brain Connect. 16, 18–35. doi: 10.1089/brain.2024.0084, 40371570

[ref11] Drobisz D. Damborská A. (2019). Deep brain stimulation targets for treating depression. Behav. Brain Res. 359, 266–273. doi: 10.1016/j.bbr.2018.11.004 30414974

[ref12] Fassi L. Hochman S. Daskalakis Z. J. Blumberger D. M. Cohen Kadosh R. (2024). The importance of individual beliefs in assessing treatment efficacy. eLife 12:89. doi: 10.7554/eLife.88889.3 PMC10977967 38547008

[ref13] Flores R. D. Sanders C. A. Duan S. X. Bishop-Chrzanowski B. M. Oyler D. L. Shim H. . (2022). Before/after Bayes: a comparison of frequentist and Bayesian mixed-effects models in applied psychological research. Br. J. Psychol. 113, 1164–1194. doi: 10.1111/bjop.12585, 35906743

[ref14] Frank D. L. Khorshid L. Kiffer J. F. Moravec C. S. Mckee M. G. (2010). Biofeedback in medicine: who, when, why and how? Ment Health Fam Med 7, 85–91, 22477926 PMC2939454

[ref15] Grogans S. E. Fox A. S. Shackman A. J. (2022). The amygdala and depression: a sober reconsideration. Am. J. Psychiatry 179, 454–457. doi: 10.1176/appi.ajp.20220412, 35775156 PMC9260949

[ref16] Hamani C. Mayberg H. Stone S. Laxton A. Haber S. Lozano A. M. (2011). The subcallosal cingulate gyrus in the context of major depression. Biol. Psychiatry 69, 301–308. doi: 10.1016/j.biopsych.2010.09.034, 21145043

[ref17] Hamilton J. P. Glover G. H. Bagarinao E. Chang C. Mackey S. Sacchet M. D. . (2016). Effects of salience-network-node neurofeedback training on affective biases in major depressive disorder. Psychiatry Res. Neuroimaging 249, 91–96. doi: 10.1016/j.pscychresns.2016.01.016, 26862057 PMC4803612

[ref18] Hamilton J. P. Glover G. H. Gotlib I. H. (2007). Healthy individuals can use real-time fmri neurofeedback to modulate activity in the subgenual anterior cingulate cortex. Biol. Psychiatry 61:30S.

[ref19] Hamilton J. P. Glover G. H. Hsu J. J. Johnson R. F. Gotlib I. H. (2011). Modulation of subgenual anterior cingulate cortex activity with real-time neurofeedback. Hum. Brain Mapp. 32, 22–31. doi: 10.1002/hbm.20997, 21157877 PMC3049174

[ref20] Hasslinger J. D'agostini Souto M. Folkesson Hellstadius L. Bölte S. (2020). Neurofeedback in Adhd: a qualitative study of strategy use in slow cortical potential training. PLoS One 15:e0233343. doi: 10.1371/journal.pone.0233343, 32497051 PMC7272030

[ref21] Haugg A. Renz F. M. Nicholson A. A. Lor C. Götzendorfer S. J. Sladky R. . (2021). Predictors of real-time fmri neurofeedback performance and improvement - a machine learning mega-analysis. NeuroImage 237:118207. doi: 10.1016/j.neuroimage.2021.118207, 34048901

[ref22] Hayes A. F. (2022). Introduction to Mediation, Moderation, and Conditional Process Analysis: A Regression-Based Approach. New York: The Guilford Press.

[ref23] Holtmann M. Steiner S. Hohmann S. Poustka L. Banaschewski T. Bölte S. (2011). Neurofeedback in autism spectrum disorders. Dev. Med. Child Neurol. 53, 986–993. doi: 10.1111/j.1469-8749.2011.04043.x, 21752020

[ref24] Hong J. Park J. H. (2022). Efficacy of neuro-feedback training for Ptsd symptoms: a systematic review and Meta-analysis. Int. J. Environ. Res. Public Health 19:96. doi: 10.3390/ijerph192013096, 36293673 PMC9603735

[ref25] Imai K. Keele L. Tingley D. Yamamoto T. Causal Mediation Analysis Using R. *In:* Vinod H. D.., ed. Advances in Social Science Research Using R, (2010) New York, Springer, 129–154.

[ref26] Johnson T. Gurubhagavatula I. (2023). Assessment of vigilance and fatigue. Sleep Med. Clin. 18, 349–359. doi: 10.1016/j.jsmc.2023.05.007, 37532374

[ref27] Johnston S. J. Boehm S. G. Healy D. Goebel R. Linden D. E. (2010). Neurofeedback: a promising tool for the self-regulation of emotion networks. NeuroImage 49, 1066–1072. doi: 10.1016/j.neuroimage.2009.07.056, 19646532

[ref28] Khaleghi A. Samiei H. Zarafshan H. Baloochi S. A. Mohammadi M. R. (2025). Effectiveness of fmri-based neurofeedback therapy on depression: a systematic review. Clin. Psychopharmacol. Neurosci. 23, 337–355. doi: 10.9758/cpn.25.1295, 40660681 PMC12264674

[ref29] Kleinbub J. R. Mannarini S. Palmieri A. (2020). Interpersonal biofeedback in psychodynamic psychotherapy. Front. Psychol. 11:1655. doi: 10.3389/fpsyg.2020.01655, 32849011 PMC7418492

[ref30] Klöbl M. Michenthaler P. Godbersen G. M. Robinson S. Hahn A. Lanzenberger R. (2020). Reinforcement and punishment shape the learning dynamics in fmri neurofeedback. Front. Hum. Neurosci. 14:304. doi: 10.3389/fnhum.2020.00304, 32792929 PMC7393482

[ref31] Klöbl M. Prillinger K. Diehm R. Doganay K. Lanzenberger R. Poustka L. . (2023). Individual brain regulation as learned via neurofeedback is related to affective changes in adolescents with autism spectrum disorder. Child Adolesc. Psychiatry Ment. Health 17:6. doi: 10.1186/s13034-022-00549-9, 36635760 PMC9837918

[ref32] Kober S. E. Buchrieser F. Wood G. (2023). Neurofeedback on twitter: evaluation of the scientific credibility and communication about the technique. Heliyon 9:e18931. doi: 10.1016/j.heliyon.2023.e18931, 37600360 PMC10432958

[ref33] Konicar L. Radev S. Prillinger K. Klöbl M. Diehm R. Birbaumer N. . (2021). Volitional modification of brain activity in adolescents with autism Spectrum disorder: a Bayesian analysis of slow cortical potential neurofeedback. Neuroimage Clin. 29:102557. doi: 10.1016/j.nicl.2021.102557, 33486138 PMC7829342

[ref34] Koush Y. Ashburner J. Prilepin E. Sladky R. Zeidman P. Bibikov S. . (2017). Opennft: An open-source Python/Matlab framework for real-time fmri neurofeedback training based on activity, connectivity and multivariate pattern analysis. NeuroImage 156, 489–503. doi: 10.1016/j.neuroimage.2017.06.039, 28645842

[ref35] Lam S. L. Criaud M. Lukito S. Westwood S. J. Agbedjro D. Kowalczyk O. S. . (2022). Double-blind, sham-controlled randomized trial testing the efficacy of fmri neurofeedback on clinical and cognitive measures in children with Adhd. Am. J. Psychiatry 179, 947–958. doi: 10.1176/appi.ajp.21100999, 36349428 PMC7614456

[ref36] Lansbergen M. M. Van Dongen-Boomsma M. Buitelaar J. K. Slaats-Willemse D. (2011). Adhd and Eeg-neurofeedback: a double-blind randomized placebo-controlled feasibility study. J. Neural Transm. (Vienna) 118, 275–284. doi: 10.1007/s00702-010-0524-2, 21165661 PMC3051071

[ref37] Li X. Hartwell K. J. Borckardt J. Prisciandaro J. J. Saladin M. E. Morgan P. S. . (2013). Volitional reduction of anterior cingulate cortex activity produces decreased cue craving in smoking cessation: a preliminary real-time fmri study. Addict. Biol. 18, 739–748. doi: 10.1111/j.1369-1600.2012.00449.x, 22458676 PMC3389595

[ref38] Linhartová P. Látalová A. Kóša B. Kašpárek T. Schmahl C. Paret C. (2019). Fmri neurofeedback in emotion regulation: a literature review. NeuroImage 193, 75–92. doi: 10.1016/j.neuroimage.2019.03.011, 30862532

[ref39] Liu S. Hao X. Liu X. He Y. Zhang L. An X. . (2022). Sensorimotor rhythm neurofeedback training relieves anxiety in healthy people. Cogn. Neurodyn. 16, 531–544. doi: 10.1007/s11571-021-09732-8, 35603045 PMC9120321

[ref40] Mehler D. M. A. Sokunbi M. O. Habes I. Barawi K. Subramanian L. Range M. . (2018). Targeting the affective brain-a randomized controlled trial of real-time fmri neurofeedback in patients with depression. Neuropsychopharmacology 43, 2578–2585. doi: 10.1038/s41386-018-0126-5, 29967368 PMC6186421

[ref41] Nadler R. T. Rabi R. Minda J. P. (2010). Better mood and better performance:learning rule-described categories is enhanced by positive mood. Psychol. Sci. 21, 1770–1776. doi: 10.1177/0956797610387441, 20974709

[ref42] Neurofeedback Collaborative Group (2021). Double-blind placebo-controlled randomized clinical trial of neurofeedback for attention-deficit/hyperactivity disorder with 13-month follow-up. J. Am. Acad. Child Adolesc. Psychiatry 60, 841–855. 32853703 10.1016/j.jaac.2020.07.906 PMC7904968

[ref43] Neurofeedback Collaborative Group (2023). Neurofeedback for attention-deficit/hyperactivity disorder: 25-month follow-up of double-blind randomized controlled trial. J. Am. Acad. Child Adolesc. Psychiatry 62, 435–446. 36521694 10.1016/j.jaac.2022.07.862 PMC10065891

[ref44] Norcross J. C. Wampold B. E. (2011). Evidence-based therapy relationships: research conclusions and clinical practices. Psychotherapy (Chic.) 48, 98–102. doi: 10.1037/a0022161, 21401280

[ref45] Peciña M. Chen J. Karp J. F. Dombrovski A. Y. (2023). Dynamic feedback between antidepressant placebo expectancies and mood. JAMA Psychiatry 80, 389–398. doi: 10.1001/jamapsychiatry.2023.0010, 36857039 PMC9979016

[ref46] Peciña M. Heffernan J. Wilson J. Zubieta J. K. Dombrovski A. Y. (2018). Prefrontal expectancy and reinforcement-driven antidepressant placebo effects. Transl. Psychiatry 8:222. doi: 10.1038/s41398-018-0263-y, 30323205 PMC6189213

[ref47] Perreau-Linck E. Lessard N. Lévesque J. Beauregard M. (2010). Effects of neurofeedback training on inhibitory capacities in Adhd children: a single-blind, randomized, placebo-controlled study. J. Neurother. 14, 229–242. doi: 10.1080/10874208.2010.501514

[ref48] Pigott H. E. Cannon R. Trullinger M. (2021). The fallacy of sham-controlled neurofeedback trials: a reply to Thibault and colleagues (2018). J. Atten. Disord. 25, 448–457. doi: 10.1177/1087054718790802, 30078340 PMC7783691

[ref49] Pigott H. E. Trullinger M. Harbin H. Cammack J. Harbin F. Cannon R. (2017). Confusion regarding operant conditioning of the Eeg. Lancet Psychiatry 4, 897–898. doi: 10.1016/S2215-0366(17)30436-4, 29179924

[ref50] Ramot M. Grossman S. Friedman D. Malach R. (2016). Covert neurofeedback without awareness shapes cortical network spontaneous connectivity. Proc. Natl. Acad. Sci. USA 113, E2413–E2420. doi: 10.1073/pnas.1516857113, 27071084 PMC4855583

[ref51] Rue H. Martino S. Chopin N. (2009). Approximate Bayesian inference for latent Gaussian models by using integrated nested Laplace approximations. J. R. Statis. Soc. Series B 71, 319–392. doi: 10.1111/j.1467-9868.2008.00700.x

[ref52] Schabus M. Griessenberger H. Gnjezda M. T. Heib D. P. J. Wislowska M. Hoedlmoser K. (2017). Better than sham? A double-blind placebo-controlled neurofeedback study in primary insomnia. Brain 140, 1041–1052. doi: 10.1093/brain/awx011, 28335000 PMC5382955

[ref53] Schönenberg M. Wiedemann E. Schneidt A. Scheeff J. Logemann A. Keune P. M. . (2017). Neurofeedback, sham neurofeedback, and cognitive-behavioural group therapy in adults with attention-deficit hyperactivity disorder: a triple-blind, randomised, controlled trial. Lancet Psychiatry 4, 673–684. doi: 10.1016/S2215-0366(17)30291-2 28803030

[ref54] Sepulveda P. Sitaram R. Rana M. Montalba C. Tejos C. Ruiz S. (2016). How feedback, motor imagery, and reward influence brain self-regulation using real-time fmri. Hum. Brain Mapp. 37, 3153–3171. doi: 10.1002/hbm.23228, 27272616 PMC6867497

[ref55] Siniatchkin M. Kropp P. Gerber W. D. (2000). Neurofeedback—the significance of reinforcement and the search for an appropriate strategy for the success of self-regulation. Appl. Psychophysiol. Biofeedback 25, 167–175. doi: 10.1023/A:1009502808906, 10999235

[ref56] Sorger B. Scharnowski F. Linden D. E. J. Hampson M. Young K. D. (2019). Control freaks: towards optimal selection of control conditions for fmri neurofeedback studies. NeuroImage 186, 256–265. doi: 10.1016/j.neuroimage.2018.11.004, 30423429 PMC6338498

[ref57] Thibault R. T. Lifshitz M. Raz A. (2016). The self-regulating brain and neurofeedback: experimental science and clinical promise. Cortex 74, 247–261. doi: 10.1016/j.cortex.2015.10.024, 26706052

[ref58] Thibault R. T. Macpherson A. Lifshitz M. Roth R. R. Raz A. (2018a). Neurofeedback with fmri: a critical systematic review. NeuroImage 172, 786–807. doi: 10.1016/j.neuroimage.2017.12.071, 29288868

[ref59] Thibault R. T. Raz A. (2017). The psychology of neurofeedback: clinical intervention even if applied placebo. Am. Psychol. 72, 679–688. doi: 10.1037/amp0000118, 29016171

[ref60] Thibault R. T. Veissière S. Olson J. A. Raz A. (2018b). Treating Adhd with suggestion: neurofeedback and placebo therapeutics. J. Atten. Disord. 22, 707–711. doi: 10.1177/1087054718770012, 29717910

[ref61] Tinaz S. Kamel S. Aravala S. S. Elfil M. Bayoumi A. Patel A. . (2022). Neurofeedback-guided kinesthetic motor imagery training in Parkinson's disease: randomized trial. Neuroimage Clin. 34:102980. doi: 10.1016/j.nicl.2022.102980, 35247729 PMC8897714

[ref62] Trambaiolli L. R. Kohl S. H. Linden D. E. J. Mehler D. M. A. (2021). Neurofeedback training in major depressive disorder: a systematic review of clinical efficacy, study quality and reporting practices. Neurosci. Biobehav. Rev. 125, 33–56. doi: 10.1016/j.neubiorev.2021.02.015, 33587957

[ref63] Trimmer P. C. Marshall J. A. R. Fromhage L. Mcnamara J. M. Houston A. I. (2013). Understanding the placebo effect from an evolutionary perspective. Evol. Hum. Behav. 34, 8–15. doi: 10.1016/j.evolhumbehav.2012.07.004

[ref64] Voigt J. D. Mosier M. Tendler A. (2024). Systematic review and meta-analysis of neurofeedback and its effect on posttraumatic stress disorder. Front. Psych. 15:1323485. doi: 10.3389/fpsyt.2024.1323485, 38577405 PMC10993781

[ref65] Watve A. Haugg A. Frei N. Koush Y. Willinger D. Bruehl A. B. . (2024). Facing emotions: real-time fmri-based neurofeedback using dynamic emotional faces to modulate amygdala activity. Front. Neurosci. 17:1286665. doi: 10.3389/fnins.2023.1286665, 38274498 PMC10808718

[ref66] Wider W. Mutang J. A. Chua B. S. Pang N. T. P. Jiang L. Fauzi M. A. . (2024). Mapping the evolution of neurofeedback research: a bibliometric analysis of trends and future directions. Front. Hum. Neurosci. 18:1339444. doi: 10.3389/fnhum.2024.1339444, 38799297 PMC11116792

[ref67] Wong C. W. Olafsson V. Tal O. Liu T. T. (2013). The amplitude of the resting-state fmri global signal is related to Eeg vigilance measures. NeuroImage 83, 983–990. doi: 10.1016/j.neuroimage.2013.07.057, 23899724 PMC3815994

[ref68] Xia Z. Yang P. Y. Chen S. L. Zhou H. Y. Yan C. (2024). Uncovering the power of neurofeedback: a meta-analysis of its effectiveness in treating major depressive disorders. Cereb. Cortex 34:252. doi: 10.1093/cercor/bhae252, 38889442

[ref69] Young K. D. Misaki M. Harmer C. J. Victor T. Zotev V. Phillips R. . (2017). Real-time functional magnetic resonance imaging amygdala neurofeedback changes positive information processing in major depressive disorder. Biol. Psychiatry 82, 578–586. doi: 10.1016/j.biopsych.2017.03.013, 28476207 PMC5610066

[ref70] Zahn R. Weingartner J. H. Basilio R. Bado P. Mattos P. Sato J. R. . (2019). Blame-rebalance fmri neurofeedback in major depressive disorder: a randomised proof-of-concept trial. Neuroimage Clin. 24:101992. doi: 10.1016/j.nicl.2019.101992, 31505367 PMC6737344

[ref71] Zhao Z. Duek O. Seidemann R. Gordon C. Walsh C. Romaker E. . (2023). Amygdala downregulation training using fmri neurofeedback in post-traumatic stress disorder: a randomized, double-blind trial. Transl. Psychiatry 13:177. doi: 10.1038/s41398-023-02467-6, 37230984 PMC10209552

[ref72] Zotev V. Phillips R. Misaki M. Wong C. K. Wurfel B. E. Krueger F. . (2018). Real-time fmri neurofeedback training of the amygdala activity with simultaneous Eeg in veterans with combat-related Ptsd. Neuroimage Clin. 19, 106–121. doi: 10.1016/j.nicl.2018.04.010, 30035008 PMC6051473

[ref73] Zweerings J. Pflieger E. M. Mathiak K. A. Zvyagintsev M. Kacela A. Flatten G. . (2018). Impaired voluntary control in Ptsd: probing self-regulation of the Acc with real-time fmri. Front. Psych. 9:219. doi: 10.3389/fpsyt.2018.00219, 29899712 PMC5989618

