# Supplementary material for: Subjective regulation success mediates the influence of neurofeedback performance on mood in the absence of training effects
Source: Front Psychol. 2026 Jul 2;17:1743425. doi: 10.3389/fpsyg.2026.1743425 (PMC13375217; doi:10.3389/fpsyg.2026.1743425)
Supplement: Supplementary file 1 [file Supplementary_file_1.docx]

Supplementary material for

Subjective regulation success mediates the influence of neurofeedback performance on mood in the absence of training effects

Klöbl M^1,2^, Robinson SD^3,4^, Silberbauer RL^1,2^, Reed MB^1,2^, Sahl A^1,2^, Gomola D^1,2^, Hahn A^1,2^, Lanzenberger R^1,2^.

^1^ Department of Psychiatry and Psychotherapy, Medical University of Vienna, Vienna, Austria

^2^ Comprehensive Center for Clinical Neurosciences and Mental Health, Medical University of Vienna, Vienna, Austria

^3^Department of Biomedical Imaging and Image-guided Therapy, Medical University of Vienna, Austria

^4^Centre for Advanced Imaging, University of Queensland, Australia

# Supplementary methods

## Study procedure

After the first NF session, subjects were given a “subject diary”, in which they could take notes on the attempted strategies and anything else about the sessions they found important. The subject diary should facilitate developing and reflecting on NF strategies but was not checked by the experimenters. During the final debriefing, subjects explained the process of finding those strategies, which resulted in the most consistent positive NF, were asked about their experience, and had the opportunity to ask any residual questions regarding the study.

## fMRI acquisition

All MRI data was recorded on a Siemens Prisma 3 T scanner. The fMRI sequence for the functional localizer, NF, and transfer run used the following parameters: echo / repetition time = 30 / 1000 ms, 2.5 mm isotropic voxels, 76 x 76 voxel matrix size, 56 slices, resulting field of view = 190 x 190 x 140 mm, multiband factor 4, bandwidth = 2120 Hz/Px. Since sgACC and amygdala are known to be particularly affected by signal dropout and artifacts in fMRI, the sequence was parametrized in order to maximize the signal-to-noise ratio while maintaining a repetition time of 1 s.

## Analysis of neurofeedback and transfer runs

Preprocessing of NF and transfer run data started with physiological noise modelling and regression using PESTICA (Beall and Lowe, 2007) followed by slice-wise motion correction and regression via SLOMOCO (Beall and Lowe, 2014). fMRI data was then slice-timing corrected, realigned across all runs within the session, distortion-corrected using topup, and realigned across sessions. Subsequent wavelet despiking (Patel et al., 2014) reduced nonlinear noise. Finally, fMRI data was normalized to the standard Space defined by the Montreal Neurological Institute (MNI) keeping the original resolution (Mueller et al., 2017) and smoothed with a Gaussian filter of 8 mm FWHM.

Each regulation block and potential reward smiley in the NF runs was modeled with an own regressor, respectively. Nuisance regression was additionally conducted via an adaptive CompCor approach (Behzadi et al., 2007, Klöbl et al., 2020). For each condition, the median contrast estimate within the respective MNI space mask derived from the functional localizer was extracted for each regulation block using the MarsBaR toolbox version 0.45 (Brett et al., 2010).

## Baseline session

The baseline session started with computerized versions of the PANAS (Breyer, 2016) and the short form of the POMS (Albani et al., 2005). The items appeared in a random order and were rated on a seven-point Likert scale from “not at all” to “extremely strong”. Subjects then entered the MRI scanner and underwent, in order, a resting-state scan (not presented here), a transfer run, the functional localizer, and a T1-weighted structural scan. This first transfer run was acquired to assess the basic ability of the subjects to volitionally influence their brain activity without prior NF training. The T1-weighted scan served as necessary anatomical reference for the NF software. After scanning, subjects again answered the PANAS and POMS. A short interview including reports on the initial strategies, motivation for participating, and expectations regarding NF concluded the baseline session (Figure 1C).

### Functional localizer

We employed a longer version of the functional localizer used in Klöbl et al. (2020), which was adapted from Hamilton et al. (2011). While the paradigm originally intended to active the sgACC, it also showed robust activation of the bilateral amygdalae rendering it suitable for the current study. The functional localizer comprised 10 blocks of three pictures shown for 6 s each with strongly negative valence from the EmoPics database (Wessa et al., 2010). These 18-s blocks of negative images were flanked by blocks of equal length in which subjects saw six commands to press and hold buttons on an MR-compatible keyboard, each time for 3 s. For details on the fMRI processing, see the supplementary section “Functional localizer analysis.” For a depiction of the selected brain regions, see Figure 1D in the main text.

### Functional localizer analysis

Unless otherwise mentioned, preprocessing and modelling of fMRI data was conducted using SPM12. Functional localizer data was slice-timing corrected and realigned, coregistered to the T1 scan, and segmented into gray matter, white matter, and cerebrospinal fluid. Conservative search masks for activation were derived from Neurosynth (neurosynth.org) association and uniformity test results for the terms “subgenual” and “amygdala”. For each term, the voxel-wise minimum of both tests was converted to Cohen’s d. These effect size maps and custom probability maps for gray matter, white matter and cerebrospinal fluid were then transformed from MNI to subject space using a pushback deformation. Brain and gray matter masks were calculated as maximum of the individual segmented and custom maps and thresholded at 50%. For white matter, cerebrospinal fluid, and a joint nuisance tissue compartment, this process was repeated using the minimum and a 95% threshold instead. SgACC and amygdalae maps were thresholded at Cohen’s d = 0.5. The functional localizer data was further preprocessed by applying the calculated brain mask, wavelet despiking, gray matter masking and Gaussian smoothing with 5 mm full with at half maximum (FWHM).

Nuisance regressors were defined following the Friston-24 model (Friston et al., 1996) and an adaptive CompCor approach. The smoothed gray matter data of the functional localizer was modeled in subject space with the negative images as active condition and button pressing as baseline. The previously calculated sgACC and amygdala masks were applied to the t-map and all voxels above the median t-value in each region selected (or all voxels with positive activation in case of a negative median) for the final individual masks. For the combined mask used in the control condition, the larger mask was limited to the size of the smaller one, selecting the respective voxels with the highest t-values before combining them, ensuring equal influence of both regions.

## Online processing and OpenNFT extensions

In order to make online processing of the NF data more comparable to offline processing, in particular CompCor and the Friston-24 model, additional artifact regression steps were included. In the beginning, only the intercept was removed. After sufficient fMRI frames were recorded, the following artifact sources were regressed out: (i) linear term, (ii) six realignment parameters and mean of the individual white matter and cerebrospinal fluid masks, (iii) realignment parameters and tissue regressors with a lag of one frame, (iv) realignment parameters and tissue regressors squared, (v) lag one realignment parameters and tissue regressors squared. All further calculations were performed as implemented in OpenNFT (Koush et al., 2017).

Feedback was presented as a moving bar over the color of the respective condition. The brightness of a circle spanning 70% of the screen followed the NF signal with additional color modulation to make changes in brightness easier to detect (red 🡪 magenta, green 🡪 yellow, blue 🡪 cyan). The modulation was exponentially down-weighted to avoid strong changes for mere fluctuations in NF. To maximize visibility of the moving bar, it was drawn in the complementary color of the modulated circle.

## Statistical modelling

### Model structure

We chose linear mixed effects models for our analysis since they allow for easy adaptation to the structure of a study and can include effects at different scales, covering, e.g., a change across NF sessions but also across the runs within a session (Zuberer et al., 2018). Learning within an operant conditioning framework is a nonlinear process (Pascual and Rodríguez, 2006) and learning curves of NF studies are particularly known to potentially be non-monotonous (Lubar et al., 1995, Dekker et al., 2014). Like in a previous work, we thus compared linear to quadratic models of regulation and outcome measures (Konicar et al., 2021). We further included crossed random effects for the color associated with each since it was shown that omitting such influences can lead to overestimated effects (Westfall et al., 2016). In addition, the reported strategies initially often showed a relationship with the color (e.g., red: “love”, green: “nature”, blue: “water”), even though subjects were informed that there is no further meaning to it. Explicitly modeling the influence of the color thus covers this observation. Additional crossed random effects for the order of conditions were included since performance might increase due to subjects becoming accustomed to the MRI scanner environment or decrease du to boredom and exhaustion. While the association with a color and order within each session can directly be modeled for the single conditions as crossed random effect (model (1): $\left( 1|col \right)+(1|ord)$), this is not possible when averaging over the conditions (model (2): $\left( 1|colAsgmt \right)+(1|ordAsgmt)$). For the latter case, we coded the six possible associations between all conditions and colors and used this assignment as random effect. Including the run number per session and condition in model (1) follows the same rationale as the order of conditions. However, the runs were modeled as fixed effects since they do not vary between subjects and sessions.

Because the resulting model structure is fairly complex, we used a Bayesian approach to fit all the random effects and reduce the risk of overfitting (Flores et al., 2022). This allowed us to implement the necessary level of complexity in the linear mixed models.

### Analysis of strategies

The center of the seven-point Likert scale was neutral indicating neither positive nor negative valence and neither arousing nor calming content. Rating was conducted only after data collection had ended and only the first author had contact to the subjects. Absolute values were used for the valence of strategies when regulating the amygdala, due to its activation to stimuli with positive as well as negative valence.

### Variable transformations

Variables with bounded range (i.e., NF / transfer run signal, PANAS / POMS scales, VAS, valence / arousal ratings) were rescaled to $\left[ 1/N,1-1/N \right]$, $N$ being the number of samples in INLA (Integrated Nested Laplace Approximations). Bounded dependent variables were then modeled using beta distributions and logit link functions. To circumvent the limitations of INLA for defining distributions and priors for independent variables, bounded independent variables were logit-transformed and mean-centered before being entered into the models.

### Moderated mediation analyses

Two exploratory models were considered to test whether the achieved regulation (represented by the NF signal) influenced the strength of mediation via subjective success ratings. The first “treatment-mediator interaction” model, permitted the achieved regulation to interact with the subjective success ratings. This was achieved by including the interaction effect between the two variables in the mediation analysis. In the second “moderated mediation” model, each run was classified as successful if a reward was received after at least 50% of the regulation blocks, and unsuccessful otherwise (see Table S1). This variable was then treated as a moderator of the mediation effect.

Due to software limitations, these analyses could not be run as mixed effects mediation models and were implemented as linear models instead. The sessions were treated as independent, with additional adjustments as detailed in the “Models” section of the main manuscript for the serial mediation analyses. The models were implemented using the “mediation” package in R and tested via the “test.TMint” and “test.modmed” functions with 1000 permutations (Imai et al., 2010).

# Supplementary results

## Demographics

Of the 25 participants who underwent NF training, scanning was unable to continue in two cases due to the participants' availability and in two other cases due to COVID restrictions. One subject withdrew from the study due to a headache after an NF session. This resulted in 20 complete datasets and five partially available ones.

## Regulation success and reward rates

As an additional indicator of training success, participants received a reward cue (a smiley face) after regulation blocks that achieved at least 10% of the maximum target regulation. A summary of the resulting reward rates is provided in Table S1. Reward rates differed across conditions, with approximately 50% of blocks rewarded during amygdala downregulation, roughly 30% during sgACC downregulation, and about 15% during simultaneous upregulation of the amygdala and sgACC (control condition).

|  | **Session** | **2** | **3** | **4** | **5** | **6** | **7** |
| --- | --- | --- | --- | --- | --- | --- | --- |
| Amy | Reward rate | 0.50 | 0.52 | 0.51 | 0.47 | 0.51 | 0.53 |
|  | Min. 50% rewards | 0.52 | 0.50 | 0.57 | 0.46 | 0.45 | 0.45 |
| sgACC | Reward rate | 0.27 | 0.32 | 0.32 | 0.28 | 0.30 | 0.27 |
|  | Min. 50% reward | 0.20 | 0.17 | 0.13 | 0.18 | 0.05 | 0.10 |
| Ctrl | Reward rate | 0.16 | 0.10 | 0.14 | 0.15 | 0.17 | 0.15 |
|  | Min. 50% rewards | 0.08 | 0.00 | 0.04 | 0.00 | 0.05 | 0.00 |

Table S1: **Reward rates across neurofeedback training sessions.** Reward rates show the fraction of blocks with sufficient regulation to trigger a reward. In addition, the fraction of participants receiving a reward in at least half of the blocks (min. 50%) for each session is shown as indicator of training success. Amy: amygdala, sgACC; subgenual anterior cingulate cortex, Ctrl: control condition.

## Partial correlation structure of mood subscales

|  |  | POMS | | | | PANAS | | |
| --- | --- | --- | --- | --- | --- | --- | --- | --- |
|  |  | anger | fatigue | vigor | POMS TMD | negative | positive | PANAS sum |
| POMS | depression | 0.32 | 0.28 | -0.27 | 0.62 | 0.65 | -0.31 | -0.46 |
|  | anger |  | 0.17 | -0.02 | 0.42 | 0.47 | -0.08 | -0.22 |
|  | fatigue |  |  | -0.45 | 0.63 | 0.31 | -0.36 | -0.37 |
|  | vigor |  |  |  | -0.63 | -0.15 | 0.61 | 0.48 |
|  | POMS TMD |  |  |  |  | 0.46 | -0.59 | -0.70 |
| PANAS | negative |  |  |  |  |  | -0.18 | -0.38 |
|  | positive |  |  |  |  |  |  | 0.91 |

Table S2: **Partial correlation structure of logit-transformed pre-training scores of the Profile of Mood States (POMS) and the Positive and Negative Affect Schedule (PANAS) adjusted for subjects and session factors.** TMD: total mood disturbance.

## Influence of pre-training mood and strategies on achieved regulation

| Condition | Measure | Variable | Estimate | 95% CI |
| --- | --- | --- | --- | --- |
| Amygdala downregulation | achieved regulation | intercept | 0.183 | 0.051, 0.315 |
|  | activation | fatigue | -0.017 | -0.032, -0.003 |
| Control (amygdala + sgACC upregulation) | activation | vigor | -0.020 | -0.033, -0.007 |
|  | achieved regulation | vigor | -0.036 | -0.069, -0.003 |
| Control (transfer, amygdala + sgACC upregulation) | activation | anger | 0.017 | 0.005, 0.028 |

Table S3: **Influence of pre-training subscales of the Profile of Mood States on brain activation and achieved regulation.** CI: credible intervals.

## Subjective ratings of achieved regulation

| Condition | Estimate | 95% CI |
| --- | --- | --- |
| Amygdala downregulation | 2.375 | 1.573, 3.166 |
| sgACC downregulation | 3.137 | 1.875, 4.384 |
| Control (amygdala + sgACC upregulation) | 2.455 | 1.203, 3.715 |

Table S4: **Influence of the achieved regulation on subjective regulation success.** CI: credible interval.

## Strategies

Participants reported a variety of strategies as successful. These include, but are not limited to, associations with the colors used to differentiate the experimental conditions (despite the information that the colors have no meaning), such as a rose for red, a forest for green, and the sea for blue; either abstract forces or mechanisms that move the bar up on the display, such as a pulling or pushing force, the bar being pulled up with a rope, or a bear pushing against the bar; exciting (e.g., bungee jumping) or relaxing (e.g., yoga) activities; natural sceneries, such as snow, the sea, a beach; emotions, such as fun, love, loneliness, stress, tension; positive events, such as a vacation, spending time with loved ones, petting a cat; sensory impressions of smell or sound; mental activities, such as mental arithmetic, focusing, or moral reasoning; abstract imaginations of, e.g., an energy field, light, emptiness, visualizing the own eye; and highly individual strategies, such as thinking about a specific video game, manga, or movie.


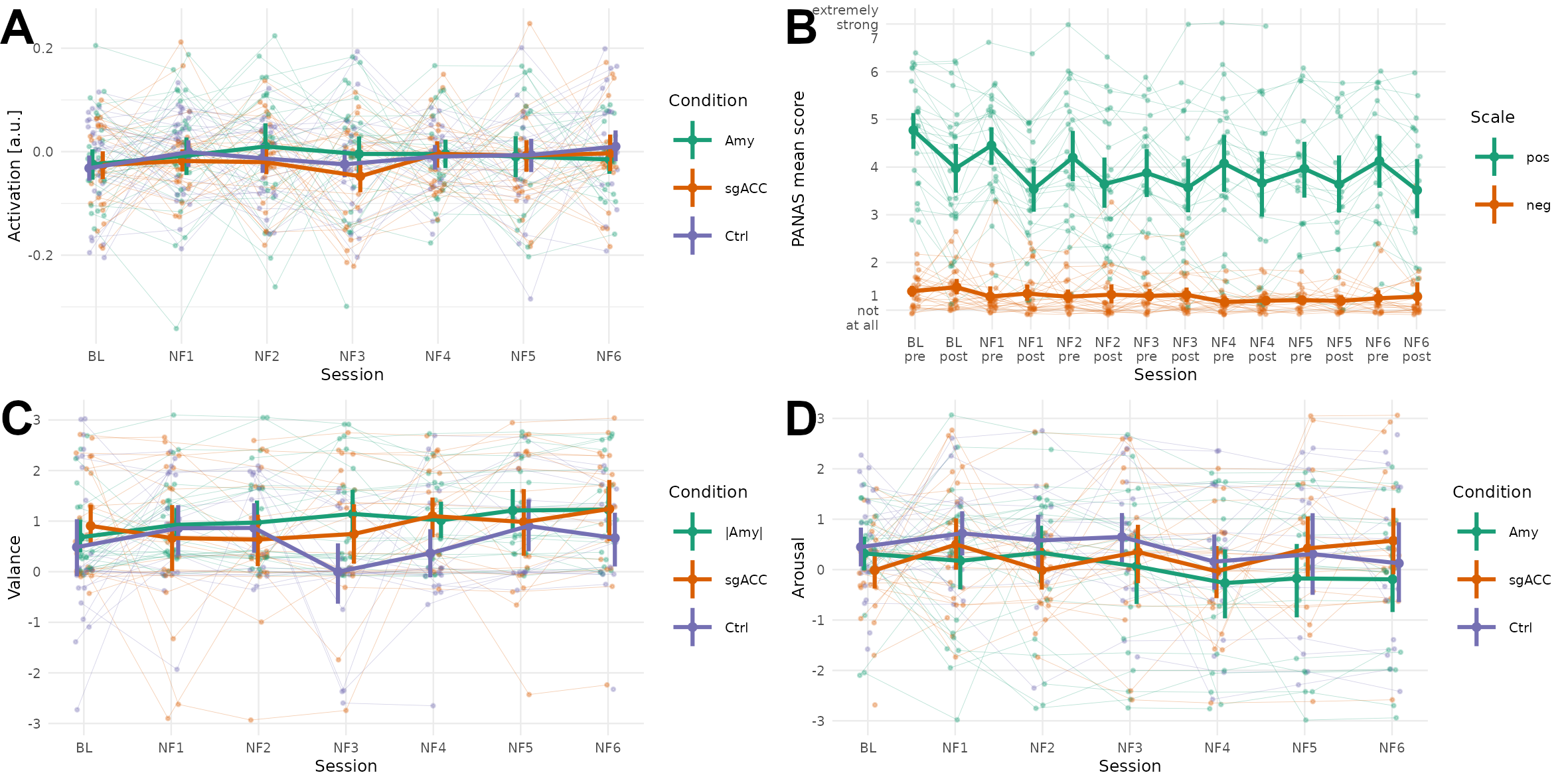


Figure S1: **Individual and mean (with 95% confidence intervals) variable time courses.** A: Transfer run activation after offline processing. B: Scales of the Positive and Negative Affect Schedule (PANAS). C: Valence of the strategies reported as most successful averaged over the three raters. The absolute value is presented for the amygdala condition like it was used in modelling. D: Arousal of the strategies reported as most successful averaged over the three raters. BL: baseline session, NF: neurofeedback session, Amy: amygdala, sgACC: subgenual anterior cingulate cortex, Ctrl: control condition.

## Mediation analyses with POMS total mood disturbance

| Condition | Effect | Estimate | 95% CI | activation direction | regulation/success direction |
| --- | --- | --- | --- | --- | --- |
| Amygdala downregulation | indirect | 0.048 | 0.009, 0.141 | ↑ | ↓ |
| sgACC downregulation | indirect | 0.033 | 0.007, 0.087 | ↑ | ↓ |

Table S5**: Significant results of serial mediation fixed effects analyses for the total mood disturbance score of the Profile of Mood States.** The indirect effects describe the full mediation path from brain activation, through achieved regulation and subjective regulation success, to the post-session scores (a_1_ * b_1_ * c in Figure 1C). CI indicates confidence intervals here. No p-values are provided for indirect effects due to potentially inappropriate t-tests. CIs not covering 0 can be interpreted as significant. Indirect effects are provided as completely standardized. sgACC: subgenual anterior cingulate cortex.

| Condition | Estimate | 95% CI | p-value |
| --- | --- | --- | --- |
| amygdala downregulation* | -0.60 | -0.99, -0.28 | < 2e-16 |
| sgACC downregulation* | -0.57 | -0.97, -0.25 | < 2e-16 |
| control (amygdala + sgACC upregulation) | -0.46 | -0.84, -0.16 | 0.0008 |

Table S 6: **Significant simple mixed mediation effects from achieved regulation over subjective regulation success to post-session total mood disturbance scores.** CI indicates confidence intervals here. * indicates confirmatory tests of serial mediation results. sgACC: subgenual anterior cingulate cortex.

## Mediation analyses with PANAS subscales

| Condition | Scale | Effect | Estimate | 95% CI | activation direction | regulation/success direction |
| --- | --- | --- | --- | --- | --- | --- |
| control (amygdala + sgACC upregulation) | positive | indirect | 0.013 | 0.001, 0.060 | ↑ | ↑ |

Table S7: **Significant results of serial mediation fixed effects analyses.** The indirect effects describe the full mediation path from brain activation, through achieved regulation and subjective regulation success, to the post-session scores (a_1_ * b_1_ * c in Figure 1C). CI indicates confidence intervals here. No p-values are provided for indirect effects due to potentially inappropriate t-tests. CIs not covering 0 can be interpreted as significant. Indirect effects are provided as completely standardized. sgACC: subgenual anterior cingulate cortex.

| Condition | Scale | Estimate | 95% CI | p-value |
| --- | --- | --- | --- | --- |
| amygdala downregulation | negative | -0.816 | -1.449, -0.280 | 0.0028 |
| sgACC downregulation | negative | -0.657 | -1.325, -0.130 | 0.0120 |
| control (amygdala + sgACC upregulation) | negative | -0.556 | -1.167, -0.110 | 0.0100 |

Table S8: **Significant simple mixed mediation effects from achieved regulation over subjective regulation success to post-session mood scores.** CI indicates confidence intervals here. sgACC: subgenual anterior cingulate cortex.

### Valence and arousal of strategies

Concordance between the independent raters was high for valence of the strategies reported as successful with a Kendall’s w = 0.88 and somewhat lower for arousal with w = 0.72.

### Moderated mediation analyses

We observed a positive treatment-mediator interaction during amygdala downregulation for the Depression subscale of the POMS questionnaire (p = 0.0264), indicating that the mediation effect was stronger during effective regulation. Moderated mediation analysis revealed a potentially positive effect of the dichotomized reward rate on the mediation pathway for the Fatigue subscale during the control condition (p = 0.0584), suggesting more positive mediation effects in runs with a higher reward rate.

# Supplementary discussion

## The interplay between mood and NF

Figures 2D and S1B demonstrate that affective scores barely change across sessions, though they exhibit a distinct sawtooth pattern before and after NF training within sessions. A very similar pattern, accompanied by changes across sessions, was also found for the POMS in patients with depression undergoing fMRI NF (Mehler, 2021). The lack of effects across sessions in our data is potentially due to floor and ceiling effects in healthy individuals. Nevertheless, NF may still induce short-term affective changes in healthy participants.

The treatment-mediator interaction and moderated mediation analyses provided some exploratory evidence suggesting that the mediation of mood changes by subjective success may be more pronounced at higher levels of regulation. While these results support the idea that subjective success drives NF-induced mood changes, the need to treat repeated sessions as independent samples due to software limitations introduced bias in the estimates and p-values. Some comparisons of simple linear models adjusted for sessions and participants with mixed effects models indicated that the former yielded overly conservative p-values (data not shown). However, we cannot conclude that this finding extends to interactions and moderated effects. Furthermore, only two combinations of NF conditions and mood subscales showed potentially meaningful effects, and their p-values would not survive multiplicity correction for the number of models. Therefore, false positive results are quite likely, which would explain why exploratory evidence for stronger mediation in the presence of higher levels of achieved regulation was only found in two unrelated cases.

## Adaptive thresholds in NF software

Since the BOLD signal has no true baseline, fMRI NF software also implicitly adapts thresholds by continuously recalculating the baseline signal to counteract drifts (Basilio et al., 2015, Koush et al., 2017), which could become detrimental if the effects of the regulation period carry over to the reference period (Pigott et al., 2017). However, this is substantially different from performance-depending adaptive thresholding. The successive regression of artifact sources employed in this study can also alter reward thresholds (supplement) but aligns with the reinforcement learning principle of shaping by encouraging more reliable strategies (Dhindsa et al., 2018).

## Strategies for the control condition

Increased activity in the amygdala and sgACC as well as increased functional connectivity between these regions has been observed in major depression *(Grogans et al., 2022, Hamani et al., 2011, Connolly et al., 2013)*. This suggests that strategies of negative valence could be used to simultaneously upregulate both regions. However, Figure S1C does not show that the strategies reported as successful for the control condition were generally less negative than those for the other conditions. Avoiding particularly negative affective strategies may have contributed to the lower performance during the control condition.

# Supplementary References

ALBANI, C., BLASER, G., GEYER, M., SCHMUTZER, G., BRÄHLER, E., BAILER, H. & GRULKE, N. 2005. [The German short version of "Profile of Mood States" (POMS): psychometric evaluation in a representative sample]. *Psychother Psychosom Med Psychol,* 55**,** 324-30.

BASILIO, R., GARRIDO, G. J., SATO, J. R., HOEFLE, S., MELO, B. R., PAMPLONA, F. A., ZAHN, R. & MOLL, J. 2015. FRIEND Engine Framework: a real time neurofeedback client-server system for neuroimaging studies. *Front Behav Neurosci,* 9**,** 3.

BEALL, E. B. & LOWE, M. J. 2007. Isolating physiologic noise sources with independently determined spatial measures. *Neuroimage,* 37**,** 1286-300.

BEALL, E. B. & LOWE, M. J. 2014. SimPACE: generating simulated motion corrupted BOLD data with synthetic-navigated acquisition for the development and evaluation of SLOMOCO: a new, highly effective slicewise motion correction. *Neuroimage,* 101**,** 21-34.

BEHZADI, Y., RESTOM, K., LIAU, J. & LIU, T. T. 2007. A component based noise correction method (CompCor) for BOLD and perfusion based fMRI. *Neuroimage,* 37**,** 90-101.

BRETT, M., ANTON, J.-L., VALABRÈGUE, R. & POLINE, J. B. Region of interest analysis using an SPM toolbox. 2010.

BREYER, B. B., M. 2016. Deutsche Version der Positive and Negative Affect Schedule PANAS.

CONNOLLY, C. G., WU, J., HO, T. C., HOEFT, F., WOLKOWITZ, O., EISENDRATH, S., FRANK, G., HENDREN, R., MAX, J. E., PAULUS, M. P., TAPERT, S. F., BANERJEE, D., SIMMONS, A. N. & YANG, T. T. 2013. Resting-state functional connectivity of subgenual anterior cingulate cortex in depressed adolescents. *Biol Psychiatry,* 74**,** 898-907.

DEKKER, M. K., SITSKOORN, M. M., DENISSEN, A. J. & VAN BOXTEL, G. J. 2014. The time-course of alpha neurofeedback training effects in healthy participants. *Biol Psychol,* 95**,** 70-3.

DHINDSA, K., GAUDER, K. D., MARSZALEK, K. A., TERPOU, B. & BECKER, S. 2018. Progressive Thresholding: Shaping and Specificity in Automated Neurofeedback Training. *IEEE Transactions on Neural Systems and Rehabilitation Engineering,* 26**,** 2297-2305.

FLORES, R. D., SANDERS, C. A., DUAN, S. X., BISHOP-CHRZANOWSKI, B. M., OYLER, D. L., SHIM, H., CLOCKSIN, H. E., MILLER, A. P. & MERKLE, E. C. 2022. Before/after Bayes: A comparison of frequentist and Bayesian mixed-effects models in applied psychological research. *Br J Psychol,* 113**,** 1164-1194.

FRISTON, K. J., WILLIAMS, S., HOWARD, R., FRACKOWIAK, R. S. & TURNER, R. 1996. Movement-related effects in fMRI time-series. *Magn Reson Med,* 35**,** 346-55.

GROGANS, S. E., FOX, A. S. & SHACKMAN, A. J. 2022. The Amygdala and Depression: A Sober Reconsideration. *Am J Psychiatry,* 179**,** 454-457.

HAMANI, C., MAYBERG, H., STONE, S., LAXTON, A., HABER, S. & LOZANO, A. M. 2011. The subcallosal cingulate gyrus in the context of major depression. *Biol Psychiatry,* 69**,** 301-8.

HAMILTON, J. P., GLOVER, G. H., HSU, J. J., JOHNSON, R. F. & GOTLIB, I. H. 2011. Modulation of subgenual anterior cingulate cortex activity with real-time neurofeedback. *Hum Brain Mapp,* 32**,** 22-31.

IMAI, K., KEELE, L., TINGLEY, D. & YAMAMOTO, T. Causal Mediation Analysis Using R. *In:* VINOD, H. D., ed. Advances in Social Science Research Using R, 2010// 2010 New York, NY. Springer New York, 129-154.

KLÖBL, M., MICHENTHALER, P., GODBERSEN, G. M., ROBINSON, S., HAHN, A. & LANZENBERGER, R. 2020. Reinforcement and Punishment Shape the Learning Dynamics in fMRI Neurofeedback. *Front Hum Neurosci,* 14**,** 304.

KONICAR, L., RADEV, S., PRILLINGER, K., KLÖBL, M., DIEHM, R., BIRBAUMER, N., LANZENBERGER, R., PLENER, P. L. & POUSTKA, L. 2021. Volitional modification of brain activity in adolescents with Autism Spectrum Disorder: A Bayesian analysis of Slow Cortical Potential neurofeedback. *Neuroimage Clin,* 29**,** 102557.

KOUSH, Y., ASHBURNER, J., PRILEPIN, E., SLADKY, R., ZEIDMAN, P., BIBIKOV, S., SCHARNOWSKI, F., NIKONOROV, A. & DE VILLE, D. V. 2017. OpenNFT: An open-source Python/Matlab framework for real-time fMRI neurofeedback training based on activity, connectivity and multivariate pattern analysis. *Neuroimage,* 156**,** 489-503.

LUBAR, J. F., SWARTWOOD, M. O., SWARTWOOD, J. N. & O'DONNELL, P. H. 1995. Evaluation of the effectiveness of EEG neurofeedback training for ADHD in a clinical setting as measured by changes in T.O.V.A. scores, behavioral ratings, and WISC-R performance. *Biofeedback Self Regul,* 20**,** 83-99.

MEHLER, D. M. A., KUNAS, STEFANIE L, SOKUNBI MOSES O, GOEBAL RAINER, LINDEN, DAVID E J 2021. Trajectories for Profile of Mood States during a multi-session neurofeedback training intervention in major depressive disorder. *PsyArXiv Preprints*.

MUELLER, K., LEPSIEN, J., MÖLLER, H. E. & LOHMANN, G. 2017. Commentary: Cluster failure: Why fMRI inferences for spatial extent have inflated false-positive rates. *Front Hum Neurosci,* 11**,** 345.

PASCUAL, M. A. & RODRÍGUEZ, M. A. 2006. Learning by operant conditioning as a nonlinear self-organized process. *Nonlinear Dynamics Psychol Life Sci,* 10**,** 341-64.

PATEL, A. X., KUNDU, P., RUBINOV, M., JONES, P. S., VÉRTES, P. E., ERSCHE, K. D., SUCKLING, J. & BULLMORE, E. T. 2014. A wavelet method for modeling and despiking motion artifacts from resting-state fMRI time series. *Neuroimage,* 95**,** 287-304.

PIGOTT, H. E., TRULLINGER, M., HARBIN, H., CAMMACK, J., HARBIN, F. & CANNON, R. 2017. Confusion regarding operant conditioning of the EEG. *The Lancet Psychiatry,* 4**,** 897.

WESSA, M., KANSKE, P., NEUMEISTER, P., BODE, K., HEISSLER, J. & SCHÖNFELDER, S. 2010. EmoPics: Subjektive und psychophysiologische Evaluation neuen Bildmaterials für die klinisch-bio-psychologische Forschung. *Zeitschrift für Klinische Psychologie und Psychotherapie,* 39**,** 77.

WESTFALL, J., NICHOLS, T. E. & YARKONI, T. 2016. Fixing the stimulus-as-fixed-effect fallacy in task fMRI. *Wellcome Open Res,* 1**,** 23.

ZUBERER, A., MINDER, F., BRANDEIS, D. & DRECHSLER, R. 2018. Mixed-Effects Modeling of Neurofeedback Self-Regulation Performance: Moderators for Learning in Children with ADHD. *Neural Plast,* 2018**,** 2464310.

# Appendix – MATLAB code

Color modulation was achieved by assigning a pattern of changes from dark to light to each of the red, green, and blue channels of the image. Depending on the assignment, the resulting color was predominantly red, green, or blue. The following code snippets show how this mechanism is implemented in OpenNFT.

% color channels

channelLRH = @(c) (c-3/8)/(3/8)*(c>=3/8&c<6/8)+(c>6/8); % low-rise-high

channelRHH = @(c) c/(3/8)*(c<3/8)+(c>=3/8); % rise-high-high

channelLLR = @(c) (c-6/8)/(2/8)*(c>6/8); % low-low-rise

% modulate sensitivity of color change to signal

colorSlope = 1.5;

% 6 ways of color assignment for 3 ROIs

switch mod(str2double(displayData.SubjectID(end-2:end)), 6)

case 0

switch roiName

case sgAccRoi % red

rChannel=channelRHH; gChannel=channelLLR; bChannel=channelLRH;

case laRoi % green

rChannel=channelLRH; gChannel=channelRHH; bChannel=channelLLR;

case ctrlRoi % blue

rChannel=channelLLR; gChannel=channelLRH; bChannel=channelRHH;

end

case 1

switch roiName

case sgAccRoi % red

rChannel=channelRHH; gChannel=channelLLR; bChannel=channelLRH;

case laRoi % blue

rChannel=channelLLR; gChannel=channelLRH; bChannel=channelRHH;

case ctrlRoi % green

rChannel=channelLRH; gChannel=channelRHH; bChannel=channelLLR;

end

case 2

switch roiName

case sgAccRoi % green

rChannel=channelLRH; gChannel=channelRHH; bChannel=channelLLR;

case laRoi % red

rChannel=channelRHH; gChannel=channelLLR; bChannel=channelLRH;

case ctrlRoi % blue

rChannel=channelLLR; gChannel=channelLRH; bChannel=channelRHH;

end

case 3

switch roiName

case sgAccRoi % green

rChannel=channelLRH; gChannel=channelRHH; bChannel=channelLLR;

case laRoi % blue

rChannel=channelLLR; gChannel=channelLRH; bChannel=channelRHH;

case ctrlRoi % red

rChannel=channelRHH; gChannel=channelLLR; bChannel=channelLRH;

end

case 4

switch roiName

case sgAccRoi % blue

rChannel=channelLLR; gChannel=channelLRH; bChannel=channelRHH;

case laRoi % red

rChannel=channelRHH; gChannel=channelLLR; bChannel=channelLRH;

case ctrlRoi % green

rChannel=channelLRH; gChannel=channelRHH; bChannel=channelLLR;

end

case 5

switch roiName

case sgAccRoi % blue

rChannel=channelLLR; gChannel=channelLRH; bChannel=channelRHH;

case laRoi % green

rChannel=channelLRH; gChannel=channelRHH; bChannel=channelLLR;

case ctrlRoi % red

rChannel=channelRHH; gChannel=channelLLR; bChannel=channelLRH;

end

end

% dispValue = [-100...100]

cIndex = (sign(dispValue)*abs(dispValue/100)^(1/colorSlope)+1)/2;

fbColor = [rChannel(cIndex) gChannel(cIndex) bChannel(cIndex)]*255;
